# Genome-Wide Analysis of Selection on the Malaria Parasite *Plasmodium falciparum* in West African Populations of Differing Infection Endemicity

**DOI:** 10.1093/molbev/msu106

**Published:** 2014-03-18

**Authors:** Victor A. Mobegi, Craig W. Duffy, Alfred Amambua-Ngwa, Kovana M. Loua, Eugene Laman, Davis C. Nwakanma, Bronwyn MacInnis, Harvey Aspeling-Jones, Lee Murray, Taane G. Clark, Dominic P. Kwiatkowski, David J. Conway

**Affiliations:** ^1^Pathogen Molecular Biology Department, London School of Hygiene and Tropical Medicine, London, United Kingdom; ^2^Medical Research Council Unit, Fajara, Banjul, The Gambia; ^3^National Institute of Public Health, Conakry, Republic of Guinea; ^4^The Wellcome Trust Sanger Institute, Hinxton, Cambridge, United Kingdom; ^5^Wellcome Trust Centre for Human Genetics, University of Oxford, Oxford, United Kingdom

**Keywords:** pathogen, balancing selection, directional selection, population genomics, immunity, transmission

## Abstract

Locally varying selection on pathogens may be due to differences in drug pressure, host immunity, transmission opportunities between hosts, or the intensity of between-genotype competition within hosts. Highly recombining populations of the human malaria parasite *Plasmodium falciparum* throughout West Africa are closely related, as gene flow is relatively unrestricted in this endemic region, but markedly varying ecology and transmission intensity should cause distinct local selective pressures. Genome-wide analysis of sequence variation was undertaken on a sample of 100 *P. falciparum* clinical isolates from a highly endemic region of the Republic of Guinea where transmission occurs for most of each year and compared with data from 52 clinical isolates from a previously sampled population from The Gambia, where there is relatively limited seasonal malaria transmission. Paired-end short-read sequences were mapped against the 3D7 *P. falciparum* reference genome sequence, and data on 136,144 single nucleotide polymorphisms (SNPs) were obtained. Within-population analyses identifying loci showing evidence of recent positive directional selection and balancing selection confirm that antimalarial drugs and host immunity have been major selective agents. Many of the signatures of recent directional selection reflected by standardized integrated haplotype scores were population specific, including differences at drug resistance loci due to historically different antimalarial use between the countries. In contrast, both populations showed a similar set of loci likely to be under balancing selection as indicated by very high Tajima’s *D* values, including a significant overrepresentation of genes expressed at the merozoite stage that invades erythrocytes and several previously validated targets of acquired immunity. Between-population *F*_ST_ analysis identified exceptional differentiation of allele frequencies at a small number of loci, most markedly for five SNPs covering a 15-kb region within and flanking the *gdv1* gene that regulates the early stages of gametocyte development, which is likely related to the extreme differences in mosquito vector abundance and seasonality that determine the transmission opportunities for the sexual stage of the parasite.

## Introduction

Evolution is driven by changing forces of selection acting upon genomes, with populations experiencing particular selective events in each generation (Olson-Manning et al. 2012). Understanding processes of adaptation requires investigation of multiple populations to identify local targets of selection, which may be similar or different across distinct populations as illustrated by studies on humans (Fu and Akey 2013; Scheinfeldt and Tishkoff 2013). Strong selection operates on malaria parasites, and their study is facilitated by a relatively small eukaryotic genome (∼23 Mb), enabling genome-wide sequence analysis of many clinical isolates of the major human parasite *Plasmodium falciparum* (Manske et al. 2012; Miotto et al. 2013).

Initial scans for evidence of positive selection on *P. falciparum* by analysis of individual endemic populations have clearly identified loci that have undergone selective sweeps, particularly from antimalarial drug use (Chang et al. 2012; Cheeseman et al. 2012; Park et al. 2012; Miotto et al. 2013; Nwakanma et al. 2014; Takala-Harrison et al. 2013), as well as loci that are apparently under balancing selection, including those encoding targets of acquired immunity (Amambua-Ngwa, Tetteh, et al. 2012). These studies have confirmed and significantly extended the findings of earlier population-genetic studies that utilized a lower density of polymorphic markers by microarray analysis (Neafsey et al. 2008; Amambua-Ngwa, Park, et al. 2012) or that focused on particular candidate loci in detail (Nash et al. 2005; Ochola et al. 2010). Such analyses have been effective for identifying loci under a single mode of strong selection, although it is likely that the direction and type of selection on many other genes is not uniform across different populations, and causes of selection aside from drugs and naturally acquired immunity have hardly been investigated. Examples of other types of selection are illustrated by considering parasite gamete surface protein genes belonging to the 6-cys family that have exceptional geographical divergence of allele frequencies (Anthony et al. 2007; Manske et al. 2012), with alleles of one of these genes (*Pfs47*) determining the ability of parasites to survive inside mosquitoes (Molina-Cruz et al. 2013).

Selection on malaria parasites will vary between locations if there are different intensities of transmission frequency or infection incidence. Parasites in highly endemic areas commonly experience within-host competition at the asexual replicating blood stage due to superinfection with different genotypes (Anderson et al. 2000), and selection for effective transmission of the sexual gametocyte stage to mosquitoes operates most of the time in such situations (Mackinnon and Read 2004). In contrast, parasites in areas of low endemicity may persist within a host without experiencing as much competition or immune selection and may only have limited opportunities for transmission due to seasonal and low-density mosquito populations. Pertinent to the present study, malaria is endemic throughout West Africa, south of the Sahara desert, but there is an extremely wide range of endemicity due to the north–south gradient in rainfall abundance and seasonality (Hay et al. 2009; Mobegi et al. 2012).

Here we report a genome-wide survey of a highly endemic *P. falciparum* population in the forest zone in south Guinea (N’Zerekore area), and comparison with a population sample previously taken from a lower transmission area in The Gambia (Ceesay et al. 2010; Amambua-Ngwa, Tetteh, et al. 2012; Nwakanma et al. 2014), to identify both shared and population-specific selective processes. The epidemiology of malaria has been less intensively studied in Guinea compared with The Gambia, so N’Zerekore was chosen for sampling as it is clearly in an area of very high endemicity with the transmission of malaria occurring for a larger part of each year compared with The Gambia (Hay et al. 2009), and a genotypic analysis with microsatellites showed that *P. falciparum* infections in N’Zerekore were much more genotypically mixed than those in The Gambia (Mobegi et al. 2012).

Findings here reveal a similar subset of genes in each population with patterns of polymorphism consistent with balancing selection, whereas there were more differences in the loci implicated as under directional selection. For example, in Guinea there is evidence of recent selective sweeps on regions containing chloroquine resistance genes *mdr1* (on chromosome 5) and *crt* (on chromosome 7); however, we observe only weak evidence of selection around the antifolate drug target *dhps* and none around *dhfr* (consistent with antifolates never having been first-line treatment in Guinea), contrasting with the history of drug use and selection in The Gambia. Further evidence of selective differences was provided by analysis of genome-wide patterns of *F*_ST_ divergence between these two closely related populations, identifying a small number of loci with extremely highly differentiated single nucleotide polymorphism (SNP) frequencies, the strongest being a cluster of SNPs on chromosome 9 within and flanking the *gdv1* gene, which plays a key role in early-stage gametocytogenesis. 

## Results

### Sequencing of *P. falciparum* and Allele Frequency Distributions of SNPs

**Fig. 1.**
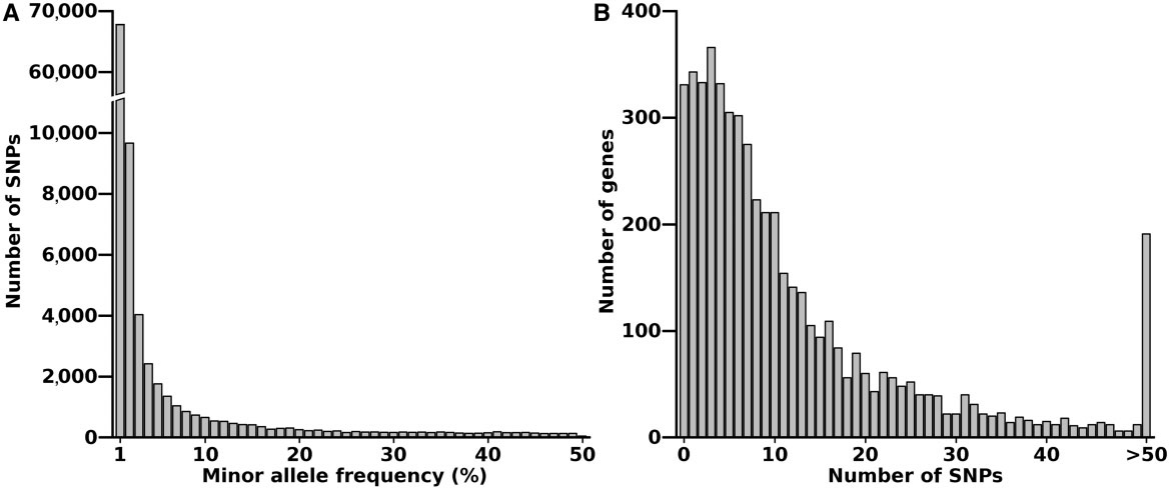
(*A*) Frequency distribution of the minor alleles for each of the SNPs scored in a population sample of 100 *P. falciparum* clinical isolates from N’Zerekore in Guinea. (*B*) Distribution of numbers of genes (*N* = 5,188 analyzed in total) with each given number of SNPs in the N’Zerekore population sample.

High-quality sequence data obtained from 100 *P. falciparum* clinical isolates collected from the N’Zerekore area of Guinea (supplementary table S1, Supplementary Material online) enabled identification of 99,305 SNPs that were polymorphic in the population. Allele calls for all isolates were present for 80,546 SNPs, with the remaining 18,759 positions missing data in <5% of the population sample. The vast majority of SNPs had a low minor allele frequency within the population, with 67,854 (68%) being observed in only a single isolate (fig. 1*A*). Coding sequences had higher read coverage compared with intergenic regions, as expected, due to less extreme A+T nucleotide richness, and as a result, 68% of all SNPs called were located within genes. Four thousand seven hundred eighty six of the 5,188 genes analyzed (subtelomeric regions along with all *var*, *rifin*, and *stevor* genes had been excluded) contained at least one SNP (fig. 1*B*). To determine whether inferences from the analyses performed in this study were unique to the population sampled in Guinea or present across West Africa, we also reanalyzed previously sampled data from a Gambian population of lower endemicity (Ceesay et al. 2010; Amambua-Ngwa, Tetteh, et al. 2012; Nwakanma et al. 2014). The Gambian population sample had 65,240 biallelic SNPs genome wide among 52 isolates using the same quality filters as applied to the Guinea population here, yielding a total of 136,144 SNPs analyzed in either population.

### Assessing the Genomic Mixedness of *P. falciparum* Infection Samples

**Fig. 2.**
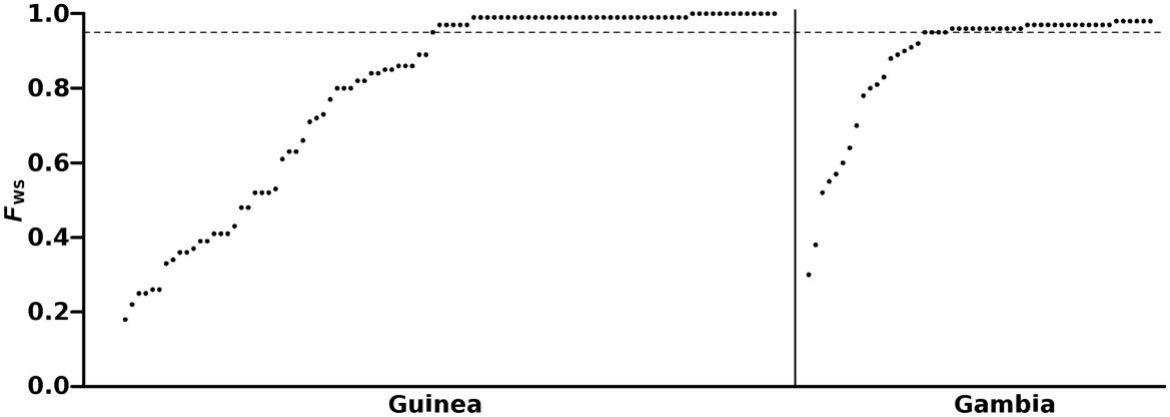
Within-infection *F*_ws_ fixation indices for each clinical isolate sampled in the Guinean and Gambian populations, ordered by increasing index value within each population. Dashed line marks *F*_ws_ = 0.95, above which an isolate may be considered to contain a single predominant genotype. The distribution of *F*_ws_ values in the Guinean population was lower than in the Gambian population (Mann–Whitney test, *P* = 0.04; *F*_ws_ values >0.95 were set at a fixed value for this comparison as they represent isolates with a single predominant genotype).

Within each sampled infection, *P. falciparum* diversity was assessed through the *F*_ws_ fixation index (Auburn et al. 2012; Manske et al. 2012), which summarizes the level of within-infection diversity (*w*) relative to that present over the whole sampled local population (*s*). In Guinea, *F*_ws_ scores of individual infections ranged from 0.18 to 1.00 (mean 0.80, median 0.97) (fig. 2), whereas values ranged from 0.30 to 0.98 in The Gambia (mean 0.88, median 0.96). An *F*_ws_ value >0.95 indicates that an infection predominantly contains a single genotype even if additional genotypes may be present at relatively low proportions, and here *F*_ws_ values >0.95 were observed for 53% and 67% of samples from Guinea and the Gambia, respectively (fig. 2 and supplementary table S2, Supplementary Material online). All subsequent population-genetic analyses were undertaken with both the whole data set and also with the subset of predominantly single genotype infections. Results were very similar, so the analyses on the complete data set are presented in the following sections (the analyses of single genotype infections are presented for comparison in the supplementary analysis file S1, Supplementary Material online).

### Identifying Signatures of Balancing Selection in Guinea

**Fig. 3.**
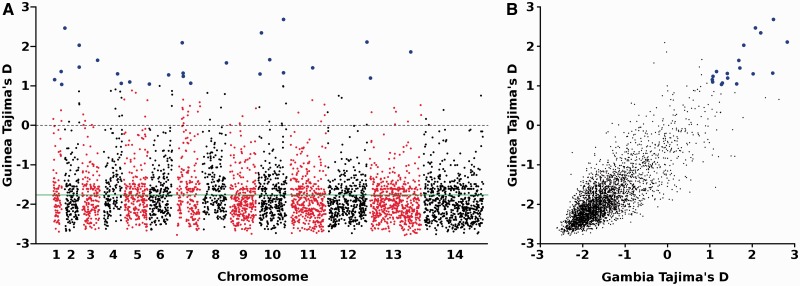
Genome-wide distribution of Tajima’s *D* values summarizing the allele frequency spectra for *P. falciparum* genes with three or more SNPs. (*A*) Tajima’s *D* values for each of 4,012 *P. falciparum* genes with three or more SNPs in Guinea (N’Zerekore population sample of 100 isolate sequences). Individual chromosomes are identified by the alternate black and red coloring, with genes plotted as individual points based on their position within each chromosome. Genes with Tajima's *D* values > 1 are highlighted with enlarged blue symbols. Detailed data for each of the genes are given in supplementary table S3, Supplementary Material online. (*B*) Correlation between Tajima’s *D* scores for the Guinea (N’Zerekore) population and a previously sampled population from The Gambia (Greater Banjul area), analyzing 3,316 genes that had three or more SNPs in each of the populations. Genes with a Tajima’s *D* value of > 1 in both populations are highlighted with enlarged blue symbols (and identified in table 1).

To study allele frequency distributions for individual genes in the Guinea population, analysis focused on the 4,012 genes that each had at least three SNPs. Tajima’s *D* values were mostly negative, with a mean of −1.76 (fig. 3*A*, supplementary table S3, Supplementary Material online), only 103 genes (2.5%) having positive Tajima’s *D* values. These predominantly negative values are consistent with previous analyses indicating a historical population expansion of *P. falciparum* in Africa (Joy et al. 2003). Three thousand three hundred sixteen genes had at least three SNPs in both Guinea and The Gambia. Across these genes, the mean Tajima’s *D* value was less negative in The Gambia (*D* = −1.44) compared with Guinea, but there was a strong correlation in Tajima’s *D* values across all genes between the two populations (fig. 3*B*, *R*^2 ^= 0.67). In terms of the top outlier genes, it is notable that 18 of the 26 genes with a Tajima’s *D* value >1 in Guinea also had a value >1 in The Gambia (fig. 3*B* and table 1), including genes previously considered most likely to be under balancing selection (Amambua-Ngwa, Tetteh, et al. 2012).

**Table 1.** Eighteen Genes with Tajima’s *D* Scores of > 1 in Both the Guinean and Gambian Populations.

Gene ID	Old Gene ID	Number of SNPs (Guinea)	Tajima’s *D* (Guinea)	Number of SNPs (Gambia)	Tajima’s *D* (Gambia)	Product Description
PF3D7_0104100	PFA0205w	65	1.16	62	1.05	Conserved plasmodium membrane protein
PF3D7_0113800	PFA0665w	230	1.36	213	1.15	DBL-containing protein
PF3D7_0114500	PFA0700c	14	1.04	14	1.27	Plasmodium-exported protein (hyp10)
PF3D7_0201600	PFB0080c	25	2.46	22	2.07	Plasmodium-exported protein (PHISTb)
PF3D7_0221000	PFB0950w	21	2.03	20	1.80	Plasmodium-exported protein
PF3D7_0321200	PFC0935c	17	1.65	15	1.68	*N*-acetylglucosamine-1-phosphate transferase, putative
PF3D7_0420200	PFD0980w	16	1.30	13	2.02	Holo-(acyl-carrier protein) synthase
PF3D7_0508800	PFE0435c	5	1.10	4	1.07	Single-stranded DNA-binding protein (SSB)
PF3D7_0601500	PFF0075c	6	1.05	5	1.64	Plasmodium-exported protein (PHISTb)
PF3D7_0710200	PF07_0042	131	1.32	118	1.41	Conserved plasmodium protein
PF3D7_0710400	MAL7P1.32	9	1.25	8	1.07	Nucleotide excision repair protein
PF3D7_0720400	PF07_0085	11	1.06	12	1.29	Ferrodoxin reductase-like protein
PF3D7_1004800	PF10_0051	18	2.34	18	2.20	ADP/ATP carrier protein
PF3D7_1035700	PF10_0348	26	1.33	21	2.48	Duffy binding-like merozoite surface protein (MSPDBL1)
PF3D7_1036300	PF10_0355	84	2.68	85	2.50	Merozoite surface protein (MSPDBL2)
PF3D7_1133400	PF11_0344	70	1.45	63	1.70	Apical membrane antigen 1 (AMA1)
PF3D7_1253100	PFL2555w	11	2.11	9	2.82	Plasmodium-exported protein (PHISTa)
PF3D7_1301800	PF13_0074, 0075	146	1.20	128	1.42	Surface-associated interspersed protein 13.1 (SURFIN 13.1)

Note.—Tajima’s *D* scores were calculated for all genes with three or more SNPs following masking or repeat regions and exclusion of SNPs within introns.

Genes with peak transcript levels at the merozoite stage in a microarray experiment of cultured parasites (Le Roch et al. 2003) had a significantly higher distribution of Tajima’s *D* values than genes with peak expression at all other stages combined (Mann–Whitney test, *P* < 10^−^^6^) or at each of the other stages individually (*P* < 0.05 for each comparison), with the exception of the late ring stage (supplementary fig. S1, Supplementary Material online). This association between stage of expression and Tajima’s *D* values for the Guinea data is similar to those obtained in a previous analysis performed on the Gambian data (Amambua-Ngwa, Tetteh, et al. 2012).

To assess whether genes associated with putative functions were enriched among the group of genes with high Tajima’s *D* values (>1.0), gene ontology (GO) term analysis was conducted. Genes associated with receptor activity (GO:0004872) and pathogenesis (GO:0009405) were found to be highly significantly enriched (*P* < 0.001) among genes with high Tajima’s *D* values in the population sample from Guinea or The Gambia. Genes annotated as having membrane-localized products were also significantly enriched among those with high Tajima’s *D* values in The Gambia (*P* = 3.9 × 10^−^^3^) or Guinea (*P* = 0.011) (supplementary table S4, Supplementary Material online).

### Detecting Signatures of Positive Directional Selection in Guinea

**Fig. 4.**
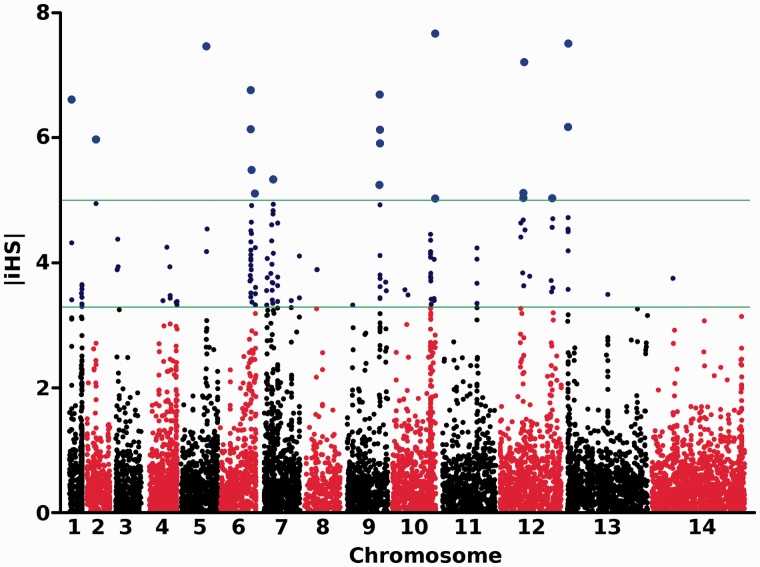
Genome-wide scan of standardized |iHS| for *P. falciparum* SNPs with minor allele frequency of at least 5% in N’Zerekore (Guinea, sequence analysis of 100 clinical isolates). Individual chromosomes are identified by alternate black and red coloring of their SNPs, with high scoring SNPs highlighted (|iHS| > 3.29 [top 1% of expected distribution] in dark blue and > 5 with enlarged light blue symbols), indicating loci most likely to have been under recent positive directional selection.

**Fig. 5.**
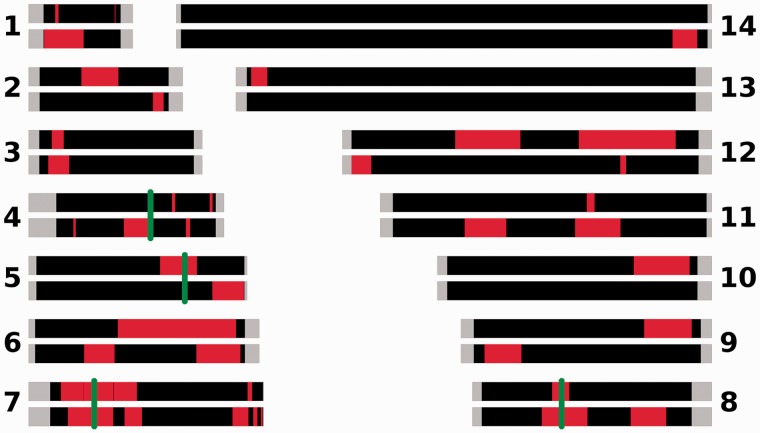
Regions of the 14 *P. falciparum* chromosomes showing signatures consistent with recent positive directional selection in the Guinea population sample (N’Zerekore) compared with the Gambian population sampled previously (Nwakanma et al. 2014). For each chromosome, the top bar represents the Guinea population, the bottom bar the Gambian population. Red shading indicates the regions containing two or more SNPs with elevated |iHS| values in either population; gray shading indicates the subelomeric regions that were not analyzed; green bars indicate the positions of antimalarial drug resistance genes *dhfr*, *mr1*, *crt*, and *dhps* on chromosomes 4, 5, 7, and 8, respectively.

We examined evidence for recent directional selection from the standardized integrated haplotype score (|iHS|) and identified 10 chromosomal loci that had two or more SNPs with a standardized |iHS| > 3.29 (top 1% of the expected distribution) and at least one SNP with an |iHS| > 5 (fig. 4 and table 2, supplementary table S5, Supplementary Material online). These identify windows containing genes that are likely to have been under exceptionally strong recent positive selection. There were strong signatures around the two major chloroquine resistance genes (*crt* on chromosome 7 and *mdr1* on chromosome 5) but not surrounding *dhfr* on chromosome 4, which confers resistance to pyrimethamine. A weak signature, involving high |iHS| values for only two SNPs, was observed around the sulphadoxine resistance gene *dhps* on chromosome 8. These results contrast with those from The Gambia, where sulphadoxine–pyrimethamine was widely used for first-line treatment, and where strong signatures of recent selection were identified around *dhfr* and *dhps* (Nwakanma et al. 2014) (fig. 5 gives a genome-wide comparison of results from the two populations).

**Table 2.** Top | iHS | Windows, Selected by the Presence of at Least a Single SNP with an | iHS | > 5 with Window Start and End Points Calculated as the Distance Required for EHH to Decay to 0.05 for SNPs with | iHS | > 3.29 (top 1% of the expected distribution).

Chromosome	Window Start (kb along Chromosome)	Window End (kb along Chromosome)	Region Size (kb)	Number of SNPs	Genes within Region
1	163	184	21	4	PF3D7_0103600-PF3D7_0104200
2	324	552	227	2	PF3D7_020800-PF3D7_0213600
**5**	**808**	**1**,**035**	**227**	**3**	**PF3D7_0519500**-**PF3D7_0524900**
6	548	1,275	727	28	PF3D7_0613500-PF3D7_0630400
**7**	**339**	**522**	**183**	**15**	**PF3D7_0707300**-**PF3D7_0711700**
9	1,126	1,419	293	16	PF3D7_0927700-PF3D7_0935800
10	1,208	1,552	344	18	PF3D7_1029700-PF3D7_1038600
12	694	1,095	401	10	PF3D7_1217600-PF3D7_1227100
12	1,454	2,050	596	7	PF3D7_1234800-PF3D7_1250100
13	93	193	101	8	PF3D7_1301600-PF3D7_1303800

Note.—Bold, windows which overlap *mdr1* and *crt* on chromosomes 5 and 7, respectively.

The genomic region containing the largest number of SNPs with a high |iHS| score in Guinea is located near one end of chromosome 6, for which a similar signature was previously observed in The Gambia (Nwakanma et al. 2014) (fig. 5) as well as in Senegal (Park et al. 2012). Highly supported windows of elevated |iHS| scores were also observed on chromosomes 9 and 10, incorporating the merozoite surface protein 1 gene (*msp1,* PF3D7_0930300) and a cluster of different antigen genes (including *GLURP*, PF3D7_1035300; and msp3, PF3D7_1035400), respectively. Although the window with elevated |iHS| containing *msp1* spans a 293-kb region, 14 of the 16 supporting SNPs are located within *msp1* itself, indicating that selection causing the signature on chromosome 9 is likely to have directly targeted MSP1.

### Genomic Scan for Differentiation between Populations in Guinea and The Gambia

Using 112,089 SNPs genome-wide for which there were no missing data, principal components analysis could not separate most isolates from the two populations. Although a small number of isolates from each population appeared as slight outliers, these were not very divergent, and the first three principal components in combination accounted for only 8.6% of total variation (supplementary fig. S2, Supplementary Material online).

**Fig. 6.**
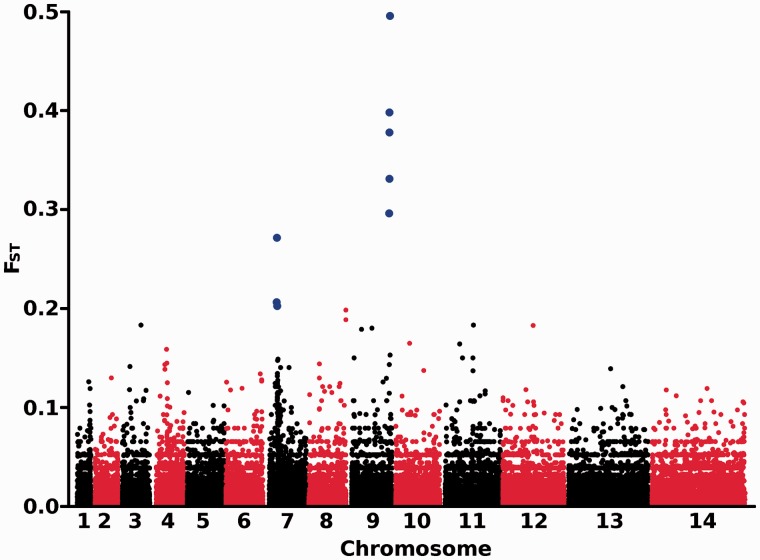
Genome-wide *F*_ST_ between the Guinean population and the Gambian population. *F*_ST_ scores were calculated for 136,144 biallelic SNPs across the genome, with each chromosome identified by the alternating black/red coloring and SNPs with *F*_ST_ > 0.2 being shown with enlarged blue symbols (table 3). The genome-wide average F_ST_ value over all SNPs was 0.0092.

The *F*_ST_ indices were analyzed for each SNP genome wide to scan for loci with exceptional allele frequency differentiation between the two populations (fig. 6). The average differentiation was very low (mean *F*_ST_ = 0.0092), consistent with the minimal genetic divergence previously estimated between these sites in analysis of microsatellite polymorphisms with independent samples (Mobegi et al. 2012), and only a few loci were highly differentiated (fig. 6 and table 3). Eight SNPs had *F*_ST_ values > 0.2, three of which are located in a ∼34-kb region of chromosome 7 within and around the major chloroquine resistance transporter locus *crt* (table 3). The five SNPs with highest *F*_ST_ values genome wide are all located within a single region of ∼15 kb on chromosome 9. One of these SNPs encodes an amino acid polymorphism within the *gdv1* gene (PF3D7_0935400) that functions to initiate early gametocytogenesis (Eksi et al. 2012), whereas the remaining four SNPs are intergenic between *gdv1* and its neighboring gene PF3D7_0935500 but closer to *gdv1*. These five SNPs are in strong linkage disequilibrium (LD) with each other (supplementary table S6, Supplementary Material online).

**Table 3.** List of the Most Highly Differentiated SNP Allele Frequencies between the Guinean and Gambian Populations.

Chromosome	SNP Position	Gene	Reference Allele Frequency (Guinea)	Reference Allele Frequency (Gambia)	*F*_ST_	Coding Effect	Amino Acid Change
7	375792	PF3D7_0708200	0.87	0.44	0.21	Synonymous	—
7	405600	PF3D7_0709000	0.87	0.37	0.27	Nonsynonymous	I → T
7	410036	PF3D7_0709100	0.77	0.31	0.20	Nonsynonymous	N → D
9	1378602	PF3D7_0935400	0.71	0.14	0.30	Nonsynonymous	P → H
9	1382170	Intergenic	0.82	0.23	0.33	—	—
9	1383344	Intergenic	0.75	0.08	0.40	—	—
9	1384752	Intergenic	0.81	0.17	0.38	—	—
9	1393934	Intergenic	0.90	0.20	0.50	—	—

Note.—*F*_ST_ scores were calculated for 136,144 biallelic SNPs genome wide (with a mean *F*_ST_ = 0.0092).

## Discussion

This population genomic study has identified parasite loci evidently under distinct processes of selection in a highly endemic population, compared with a population of relatively low endemicity within the same geographical region, as well as loci that are apparently under similar selective processes. It is advantageous to apply genome-wide sequence analyses at population level to study natural selection in African populations of *P. falciparum* as the parasite has a high rate of recombination, large effective population size, and high rates of gene flow throughout the region, particularly in West Africa (Manske et al. 2012; Mobegi et al. 2012; Miotto et al. 2013). Furthermore, known differences in historical drug selection provide a type of control for the interpretation of results as reflecting signatures of selection (Nwakanma et al. 2014).

Malaria transmission intensity and parasite genetic diversity are known to vary greatly among different parts of West Africa due to variation in rainfall abundance and seasonality, and microsatellite studies have clearly indicated more highly mixed genotype infections in Guinea than in an area of lower transmission in The Gambia (Mobegi et al. 2012). Analysis of within-infection diversity in a genome-wide study of SNPs supports this and also indicates that multiple genotype infections often contain a predominant genotype at the time of sampling, with other SNP alleles from additional genotypes being at very low frequency within the infection. The presence of multiple genotype infections could compromise haplotype-based tests of selection due to the possibility of constructing false haplotypes when scoring the predominant allele at each SNP within such infections, but analysis of the subset of single predominant genotype infections here showed similar results to analysis of the whole population sample.

The existence of extended haplotypes at high frequencies demonstrated selective sweeps occurring around the chloroquine resistance genes *crt* and *mdr1* in Guinea, consistent with the use of chloroquine alone in first-line treatment for malaria until 2006 when the amodiaquine–artesunate combination was recommended and began to gradually replace it. In contrast, we did not detect evidence of selection associated with the resistance gene *dhfr* in Guinea and observed only a weak signature around *dhps*, as the combination sulphadoxine–pyrimethamine that targets these gene products was never introduced as a first-line treatment in this country. A positive control comparison was provided by signatures of selection at these loci in The Gambia, reflecting the therapeutic use of sulphadoxine–pyrimethamine in that country until 2008 (Nwakanma et al. 2014). Genome wide, most of the regions of high |iHS| are particular to one or other of the populations, suggesting that there is spatially varying selection on other loci apart from drug resistance genes. However, there are a few examples of shared |iHS| regions, including the *crt* locus on chromosome 7, and most notably, a large region of chromosome 6 for which a similar result has also been reported from Senegal (Park et al. 2012). It is not clear what the mechanism of selection has been on the chromosome 6 locus, as analysis of Senegalese samples suggested a potential association with pyrimethamine resistance (Park et al. 2012), but it is unlikely that pyrimethamine caused very strong selection in Guinea, where it has not been officially part of first-line therapy for malaria and no selective signature was seen for the *dhfr* gene. It is notable that a high |iHS| score was associated with the gene encoding the MSP1 antigen on chromosome 9, as this gene has a complex pattern of polymorphism that is likely to result from different selective processes. Evidence of balancing selection has been seen for a highly polymorphic N-terminal “block 2” region which is a target of allele-specific immunity (Conway et al. 2000), but most of the rest of the coding sequence has two highly divergent allelic types between which there is a complete LD (Tanabe et al. 2007). These major dimorphic types exist at geographically varying frequencies (Conway 1997) that have been shown to be highly skewed but temporally stable in The Gambia (Conway et al. 1992), but a full interpretation of the |iHS| score may require further analysis of apparent heterogeneity in recombination rate occurring between allelic variants within each of the major types (Tanabe et al. 2007). Similarly, there may be complex processes of balancing and directional selection on the chromosome 10 cluster of genes encoding antigens such as MSP3 and GLURP, and allele type-specific recombination rates could be considered in exploring the basis of the observed high |iHS| values further in this genomic region.

Allele frequency distributions indicating the operation of balancing selection were evident in a similar subset of genes in Guinea as in The Gambia. This is consistent with expectations that balancing selection due to allele frequency-dependent acquired immune responses is likely to operate on similar antigenic targets in both populations, even though the intensity of immune selection is likely to be higher in Guinea. Genes showing the highest values of Tajima’s *D* in both populations, consistent with strong balancing selection include those encoding known antigens such as AMA1, MSP3, MSPDBL1, MSPDBL2, as well as those that encode probable targets of immunity that require further study (other DBL-containing proteins and members of the SURFIN and PHIST families), while several other genes encoding vaccine candidate antigens had moderately positive values of Tajima’s *D* (supplementary table S3, Supplementary Material online). Particular antigen genes have shown consistent evidence indicating balancing selection within different sampled populations (Ochola et al. 2010; Weedall and Conway 2010), and the analysis of the Guinea population essentially reinforces the identification of loci most likely to be under balancing selection in an earlier analysis of the Gambian population (Amambua-Ngwa, Tetteh, et al. 2012).

The most extreme allele frequency divergence between the populations was seen in a 15-kb region of chromosome 9 that includes a single gene (*gdv1*) encoding the gametocyte development 1 protein (Eksi et al. 2012). This protein plays a key role in development, regulating the induction of early differentiation into gametocytes, and the gene has been spontaneously as well as purposefully deleted from several laboratory lines that have thereby lost the ability to produce gametocytes in culture (function is restored through complementation by *gdv1*) (Eksi et al. 2012). It is possible that the different alleles that show high fixation between the Guinea and Gambia mediate a different response to environmental triggers or a different baseline rate of switching to gametocytes, as there is transmission for most of the year in Guinea but only seasonally in The Gambia. The reference (matching the 3D7 genome) allele, which is predominant in Guinea, is present at high frequencies in genome sequence data from other populations with high levels of malaria transmission in Burkina Faso, Ghana, and southern Mali, while a lower frequency exists in Senegal where there is more moderate malaria endemicity, and this allele appears to be completely absent within Southeast Asia, where malaria is generally less endemic than in Africa (Chang et al. 2012; Manske et al. 2012; Miotto et al. 2013; Preston et al. 2014). Induction of gametocytogenesis is likely to involve numerous modifiers (Baker 2010), but genetic manipulation experiments by parasite transfection may identify causal allelic determinants in the *gdv1* gene region.

The signature of differentiation of allele frequencies at the *crt* locus reflects differences in the intensity and timing of selection by chloroquine in the two populations, as the resistance allele frequency is highly labile and declines due to fitness costs after the use of chloroquine has ceased (Kublin et al. 2003; Nwakanma et al. 2014). Indeed, this was the locus that most clearly showed signatures of recent directional selection within each population (extremely high |iHS| values) as well as exceptional differentiation between populations (extremely high *F*_ST_ values). Identification of genes under more moderate processes of differential selection locally is likely to be most effectively achieved by genome-wide analysis of additional populations to build up relevant data sets for pairwise and matrix analyses. This is warranted as the control of major infectious diseases such as malaria requires intensive efforts, which should be guided by a thorough understanding of adaptive processes occurring in pathogen populations in different endemic areas.

## Materials and Methods

### Ethics Statement

Permission to conduct the collection and analysis of clinical samples was granted by the Comite d’Ethique National Pour la Recherché en Santé, Republique de Guinee (National Ethics Committee for Health Research, Republic of Guinea) following review of the proposed research. Written informed consent was obtained from a parent or guardian of each child included in the study, and locally authorized treatment for malaria with Artesunate-Amodiaquine was provided regardless of inclusion in the study.

### Sampling of *P. falciparum* Parasites from Malaria Patients

Malaria patients were sampled from local health facilities located within 25 km of the regional hospital in N’Zerekore, Republic of Guinea between March and May 2011. Patients were eligible for recruitment if they were children more than 1-year old presenting with an axillary temperature of > 37.5 °C or history of fever within the last 48 h. After consent, detection of *P. falciparum* malaria parasites was performed by rapid diagnostic test (Paracheck, Orchid Biomedical systems, India), and a venous blood sample of up to 5 ml was requested from each patient that had a parasite positive test. Blood was collected in ethylenediaminetetraacetic acid vacutainers, depleted of leukocytes using a standard protocol of filtration through CF11 cellulose columns (Venkatesan et al. 2012), and then frozen at −20 °C. Thick and thin blood films were prepared from each blood sample before and after leukocyte depletion. Samples were considered suitable for DNA extraction if microscopic examination of the thick blood films indicated that leukocytes had been removed, and the thick and thin blood films clearly showed *P. falciparum* in the absence of other detectable parasite species. Frozen blood and slides were transported to the MRC Laboratories in The Gambia for extraction of DNA using the QIAamp blood midi kit (Qiagen, UK) and confirmation of *P. falciparum* parasitaemia.

### Whole-Genome Sequencing of *P. falciparum* from Clinical Isolates

DNA preparations extracted from 140 leukocyte-depleted clinical samples confirmed to contain *P. falciparum* underwent quality control screening before sequencing. For 132 (94%) of the samples, the amount and purity of *P. falciparum* DNA was above minimal levels recommended by the sequencing pipeline at the Wellcome Trust Sanger Institute, so sequencing proceeded on the Illumina HiSeq platform using previously developed protocols (Manske et al. 2012; Miotto et al. 2013). Sequence read data obtained for each isolate are available through the European Nucleotide Archive (accession details listed in supplementary table S1, Supplementary Material online). Reads were mapped to the *P. falciparum* 3D7 reference sequence (v3, October 2012) using SMALT (http://www.sanger.ac.uk/resources/software/smalt/, last accessed November 5, 2013) with default parameters, and SNPs were called using SAMTOOLS as applied previously to a Gambian data set (Nwakanma et al. 2014). For each SNP, the majority allele within each infection was counted toward analyses of population allele frequencies. Analyses were subsequently conducted on all infection samples and also on the subset of infections that were least mixed and apparently contained a single predominant genotype as assessed by the *F*_WS_ analysis described below. SNPs were excluded from analysis if they were positioned within subtelomeric regions (supplementary table S7, Supplementary Material online), if they were located within the hypervariable *var*, *rifin*, and *stevor* gene families, or were positioned within repetitive sequences as identified by Tandem Repeat Finder (Settings: match: 2, mismatch: 7, delta: 7, pM: 80, pI: 10, minscore: 40, max-period: 500). Data were then filtered to exclude isolates and SNP positions with excessive missing calls (isolates with >10% missing SNPs, and SNPs with >5% missing isolate data). The filtered population data set for the N’Zerekore population consisted of 100 isolates and 99,305 biallelic SNPs, with allele calls for each isolate available for 80,546 SNPs.

Sequence data from the previously studied population from the Greater Banjul area of The Gambia (Amambua-Ngwa, Tetteh, et al. 2012; Nwakanma et al. 2014) were reanalyzed from the original paired-end short reads, to provide a standardized comparison with the new data presented here from N’Zerekore in Guinea. After filtering, the combined data set for analysis comprised 100 isolates from Guinea, and 52 from The Gambia, with a total of 136,144 genome-wide biallelic SNPs.

### Population Genetic Tests

Within-host diversity was assessed through the *F*_WS_ metric, calculated as previously described (Manske et al. 2012). For all biallelic genic SNPs, within isolate expected heterozygosity values (*H*_w_) were calculated from the relative allele frequencies and compared with the local population heterozygosity (*H*_s_), to derive *F*_ws_ = (*H*_s_ − *H*_w_/*H*_s_). For this analysis, individual alleles with a coverage of <5 reads and positions with a total coverage of <20 reads were classified as missing data. Isolates with >20% missing SNP data and SNPs with >10% missing isolate data were discarded, producing a final set of 54,175 Guinean and 33,290 Gambian SNPs. Isolates with *F*_ws_ scores of >0.95 were classed as having a single predominant genotype due to limited genome-wide diversity, with this subset used to assess whether the whole population analysis was affected by the inclusion of diverse complex infections.

Analyses of allele frequency distributions, within-population Tajima’s *D* indices (Tajima 1989), and between-population *F*_ST_ values (Weir and Cockerham 1984) were calculated using custom R scripts. For Tajima’s *D* analysis, missing data were observed to cluster in subsets of isolates at each gene and were, therefore, excluded on a per gene basis by removal of those isolates. For *F*_ST_ analysis, missing data were excluded on a per SNP basis with the size of each population corrected to account for the removal of isolates. LD was calculated using the Genetic Distance Analysis program (GDA; http://www.eeb.uconn.edu/people/plewis/software.php, last accessed November 5, 2013). Signatures of positive directional selection in the Guinea population were identified using the standardized |iHS|, which was calculated for each SNP with no missing data and a minor allele frequency of >0.05 (Voight et al. 2006), as has been previously applied to the Gambian population sample (Nwakanma et al. 2013). The genetic distance between each SNP was inferred with LDhat (McVean et al. 2002), using a block penalty of 5, 10 million rjMCMC iterations, and a burn in of 100,000 iterations. Selection windows were defined by calculating the distance required for the extended haplotype homozygosity of each SNP to decay to a level of 0.05 in each direction using the SWEEP program (Sabeti et al. 2002). Overlapping EHH windows from individual high-scoring SNPs (|iHS| > 3.29) were combined into continuous windows, and windows supported by only a single SNP position were subsequently discarded.

Expression time-series query in PlasmoDB (Aurrecoechea et al. 2009) was used to assign the parasite stage of peak expression in culture as determined by microarray studies (Le Roch et al. 2003) on all genes for which a Tajima’s *D* score was calculated for the Guinea population (both stage of peak expression and Tajima’s *D* score was available for 3,807 genes). Median values of Tajima’s *D* scores for the set of genes with an expression peak at each stage were calculated, and Mann–Whitney tests were used to assess the significance of pairwise differences between the Tajima’s *D* scores for genes grouped by stage of peak expression.

Genes with a Tajima’s *D* value >1.0 were classed as genes of potential interest for GO analysis. Analysis was performed using TopGO (R package version 2.10.0, http://www.bioconductor.org/, last accessed November 5, 2013). *P* values were calculated using Fisher’s exact test and adjusted to account for the GO graph topology using the weight algorithm proposed previously (Alexa et al. 2006).

## Supplementary Material

Supplementary analysis file S1, tables S1–S3, and figures S1 and S2 are available at *Molecular Biology and Evolution* online (http://www.mbe.oxfordjournals.org/).

Supplementary Data
