# Supplementary material for: Genome-Wide Analysis of Selection on the Malaria Parasite Plasmodium falciparum in West African Populations of Differing Infection Endemicity
Source: Mol Biol Evol. 2014 Mar 18;31(6):1490–9. doi: 10.1093/molbev/msu106 (PMC4032133; doi:10.1093/molbev/msu106)
Supplement: Supplementary Data [file supp_31_6_1490__index.html]

Genome-Wide Analysis of Selection on the Malaria Parasite Plasmodium falciparum in West African Populations of Differing Infection Endemicity — Genome-Wide Analysis of Selection on the Malaria Parasite Plasmodium falciparum in West African Populations of Differing Infection Endemicity — Supplementary Data 

# Genome-Wide Analysis of Selection on the Malaria Parasite *Plasmodium falciparum* in West African Populations of Differing Infection Endemicity

## Supplementary Data

files

**Files in this Data Supplement:**

- Supplementary Data - pdf file
- Supplementary Data - pdf file
- Supplementary Data - pdf file
- Supplementary Data - xlsx file
