# Supplementary material for: Genome-Wide Analysis of Selection on the Malaria Parasite Plasmodium falciparum in West African Populations of Differing Infection Endemicity
Source: Mol Biol Evol. 2014 Mar 18;31(6):1490–9. doi: 10.1093/molbev/msu106 (PMC4032133; doi:10.1093/molbev/msu106)
Supplement: Supplementary Data [file supp_msu106_Supplementary_Analysis_File.pdf]

Supplementary Analysis

A subset of *P. falciparum* infections that each contained predominantly a single genotype were identified in both the Guinea and The Gambia populations using the F_{ws} metric, with scores of > 0.95 indicating that a single parasite genotype accounted for the majority of observable reads within the isolate (Figure 2, Supplementary Table S2). The predominantly single genotype populations consisted of 50 Guinean and 33 Gambian isolates and 106009 biallelic SNPs. In order to ensure mixed genotype infections had not had a negative impact on the accuracy of allele calls the primary analysis was repeated in full using only those isolates identified as being predominantly single genotype, the results of which are presented below and which show close general agreement with the analysis of the complete 152 isolate dataset presented in the main paper.

Supplementary Analysis Figure I. A. Frequency distribution of the minor alleles for each of the SNPs scored in a population sample of 50 predominantly single genotype *P. falciparum* isolates from N'Zerekore in Guinea. **B.** Distribution of numbers of genes (N = 5188 analysed in total) with each given number of SNPs in the N'Zerekore population sample.

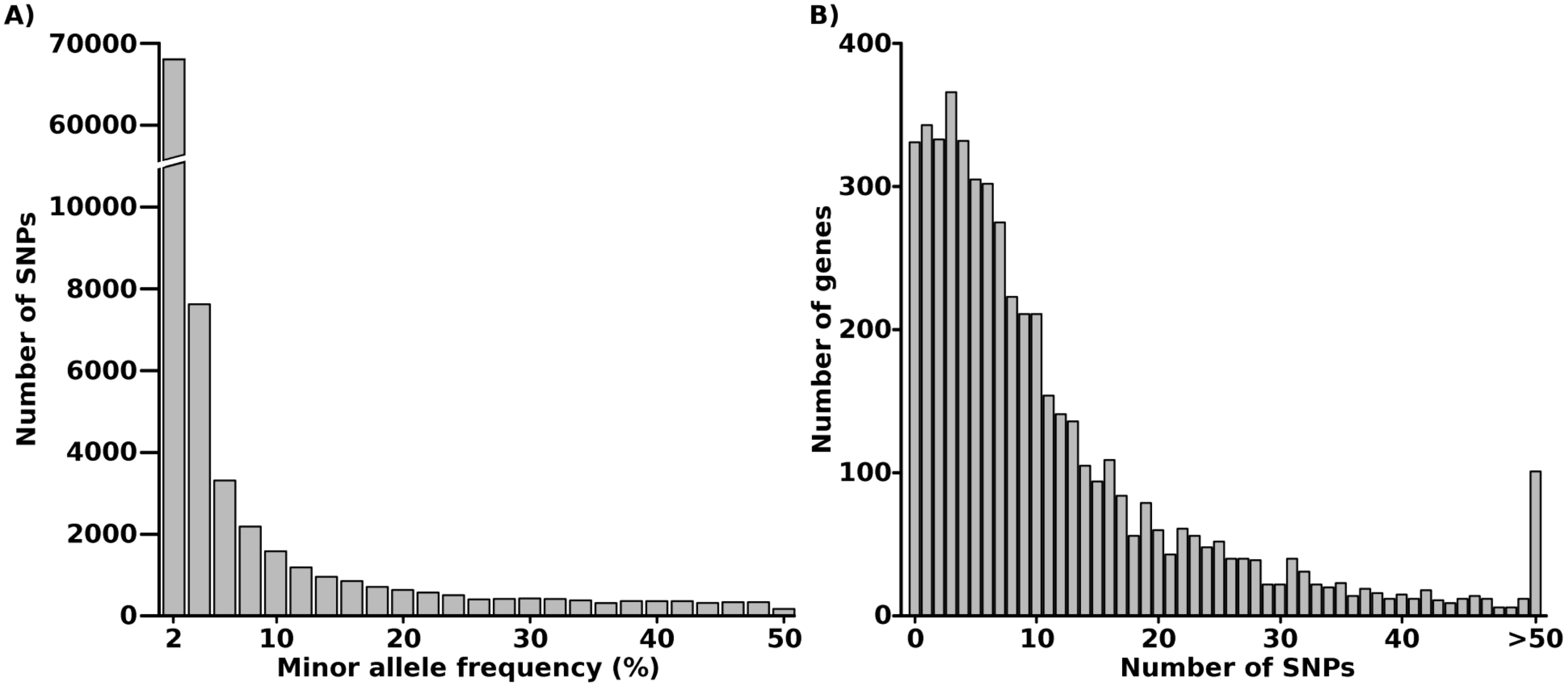

Supplementary Analysis Figure II. Genome wide distribution of Tajima's D values summarising the allele frequency spectra for *P. falciparum* genes with 3 or more SNPs. **A.** Tajima's D values for each of 3696 *P. falciparum* genes with 3 or more SNPs in Guinea (N'Zerekore predominantly single genotype population sample of 50 isolate sequences). Individual chromosomes are identified by the alternate black and red colouring, with genes plotted as individual points based on their position within each chromosome. Detailed data for each of the genes are given in Supplementary Analysis Table IV. **B.** Correlation between Tajima's D scores for the Guinea (N'Zerekore) population and a previously sampled population from The Gambia (Greater Banjul area), analysing 2955 genes that had 3 or more SNPs in each of the populations. Genes with a Tajima's D value of > 1 in both populations are highlighted in blue (and identified in Table 1).

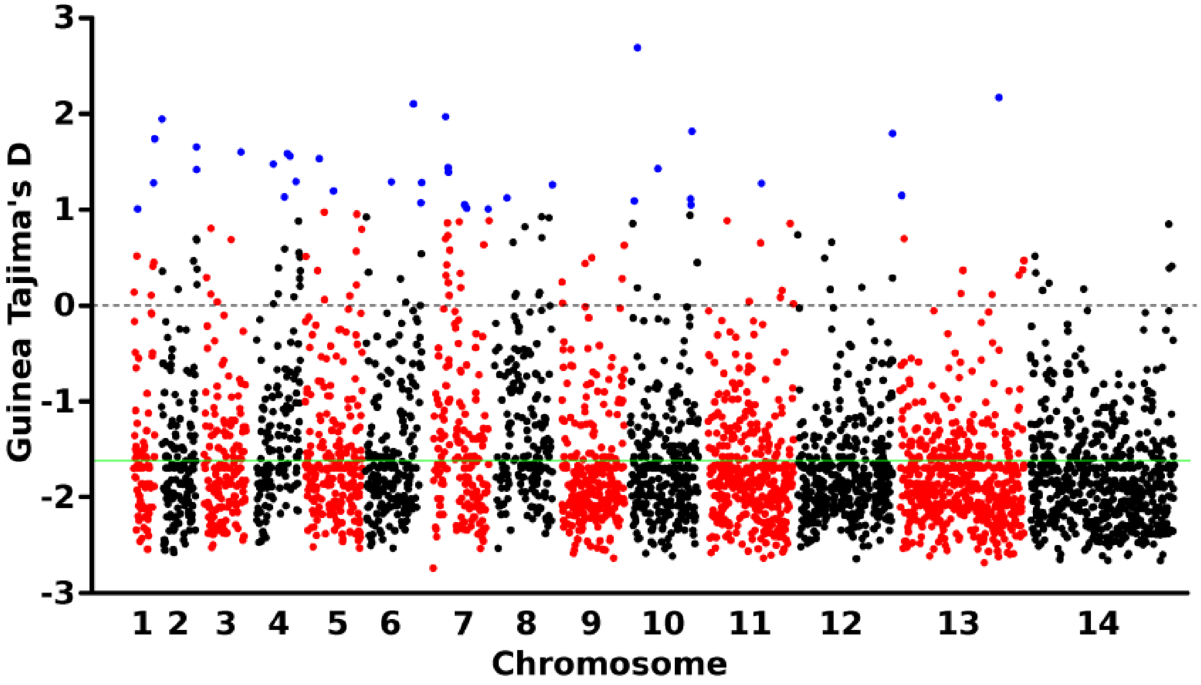

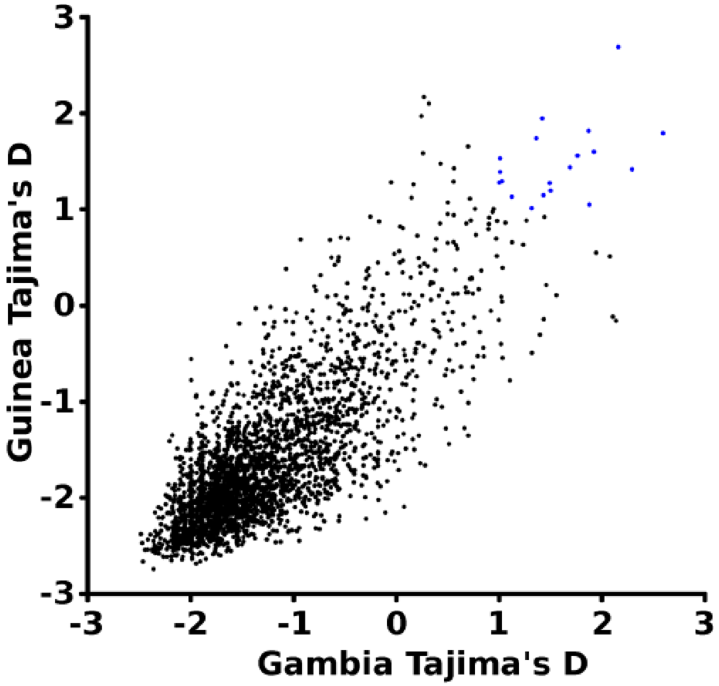

Supplementary Analysis Figure III. Genome wide scan of standardised integrated haplotype scores (|iHS|) for *P. falciparum* SNPs with minor allele frequency of at least 5% in N'Zerekore (Guinea, sequence analysis of 50 predominantly single genotype clinical isolates). Individual chromosomes are identified by alternate black and red colouring of their SNPs, with high scoring SNPs highlighted (|iHS| > 3.29 [top 1% of expected distribution] in dark blue and > 5 in light blue) indicating loci most likely to have been under recent positive directional selection.

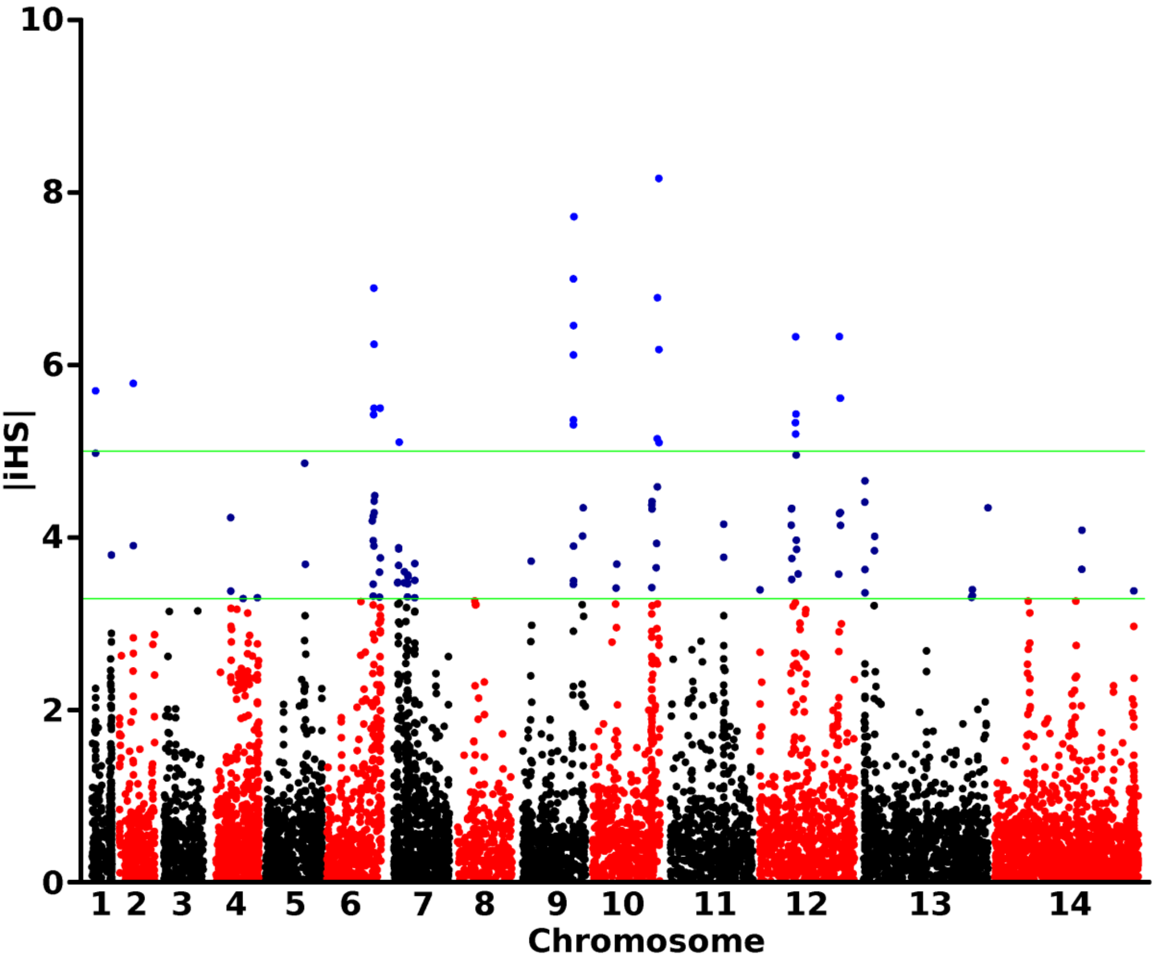

Supplementary Analysis Figure IV. Regions of the 14 *P. falciparum* chromosomes showing signatures consistent with recent positive directional selection in the predominantly single genotype Guinea population sample (N'Zerekore) compared with the Gambian population sampled previously (Nwakanma et al. 2013). For each chromosome the top bar represents the Guinea population, the bottom bar the Gambian population. Red shading indicates the regions containing 2 or more SNPs with elevated |iHS| values in either population; grey shading indicates the sub-telomeric regions that were not analysed; green bars indicate the positions of antimalarial drug resistance genes *dhfr*, *mr1*, *crt* and *dhps* on chromosomes 4, 5, 7 and 8 respectively.

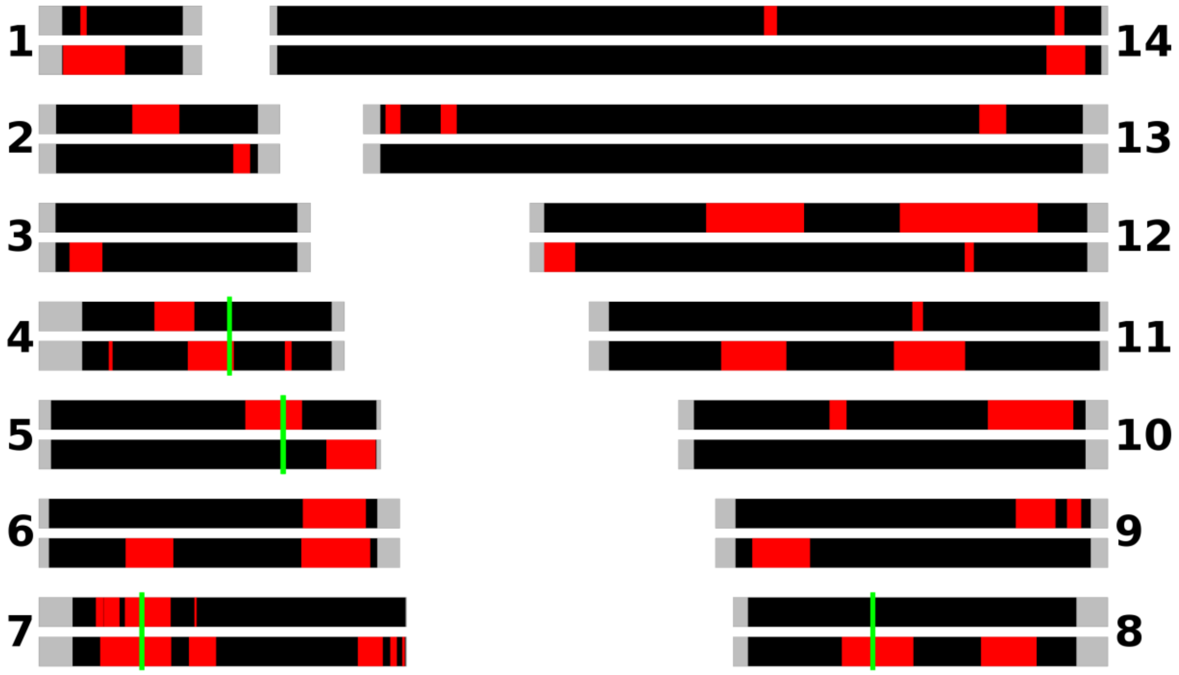

Supplementary Analysis Figure V. Genome wide F_{ST} between the 2011 Guinean and the 2008 Gambian predominantly single genotype populations. F_{ST} scores were calculated for 106009 biallelic SNPs across the genome, with each chromosome identified by the alternating black / red colouring and SNPs with F_{ST} > 0.2 being shown in blue (Supplementary Analysis Table III).

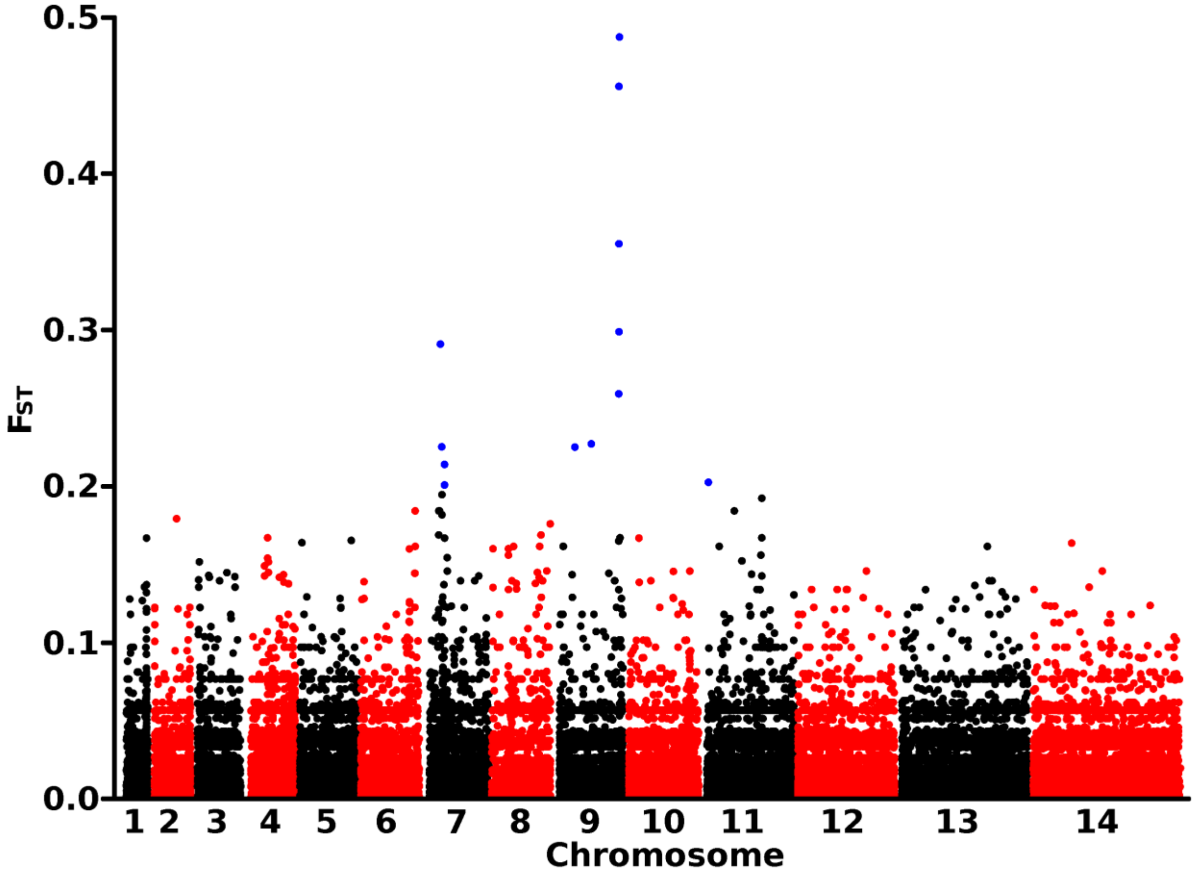

Supplementary Analysis Figure VI. Tajima's D value distribution is highest for genes predicted to have peak expression at the merozoite stage. Expression data for 3523 genes from microarray studies (Le Roch et al. 2003) retrieved from PlasmoDB (Aurrecoechea et al. 2009) were used to group genes by stage of peak expression. Points show the Tajima's D score for each gene in the Guinean population (grey bars show the medians for all genes with the same stage of peak expression) with predicted peak expression at each stage in the erythrocytic life cycle (ER, early ring; LR, late ring; ET, early trophozoite; LT, late trophozoite; ES, early schizont; LS, late schizont; M, merozoite). Asterisks indicate p values given by Mann-Whitney tests for the merozoite stage versus each other stage individually (* p<0.05, *** p<0.0001).

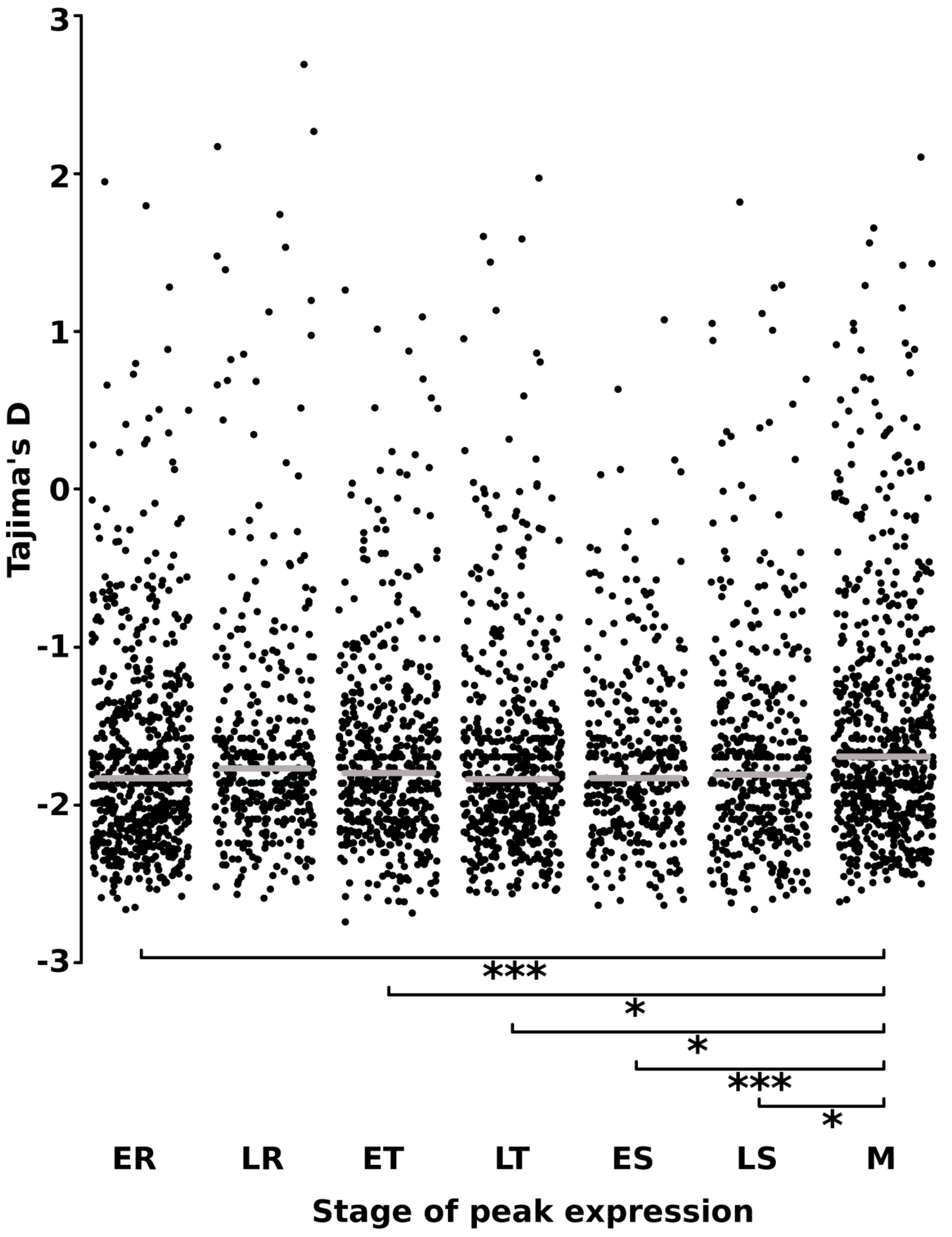

Supplementary Analysis Figure VII. Principal component analysis of 83 predominantly single genotype isolates from Guinea and Gambia utilising 112089 SNPs. A. First component and second component (3.5% and 3.4% of total variation respectively). B. Second and third component (3.4% of variation).

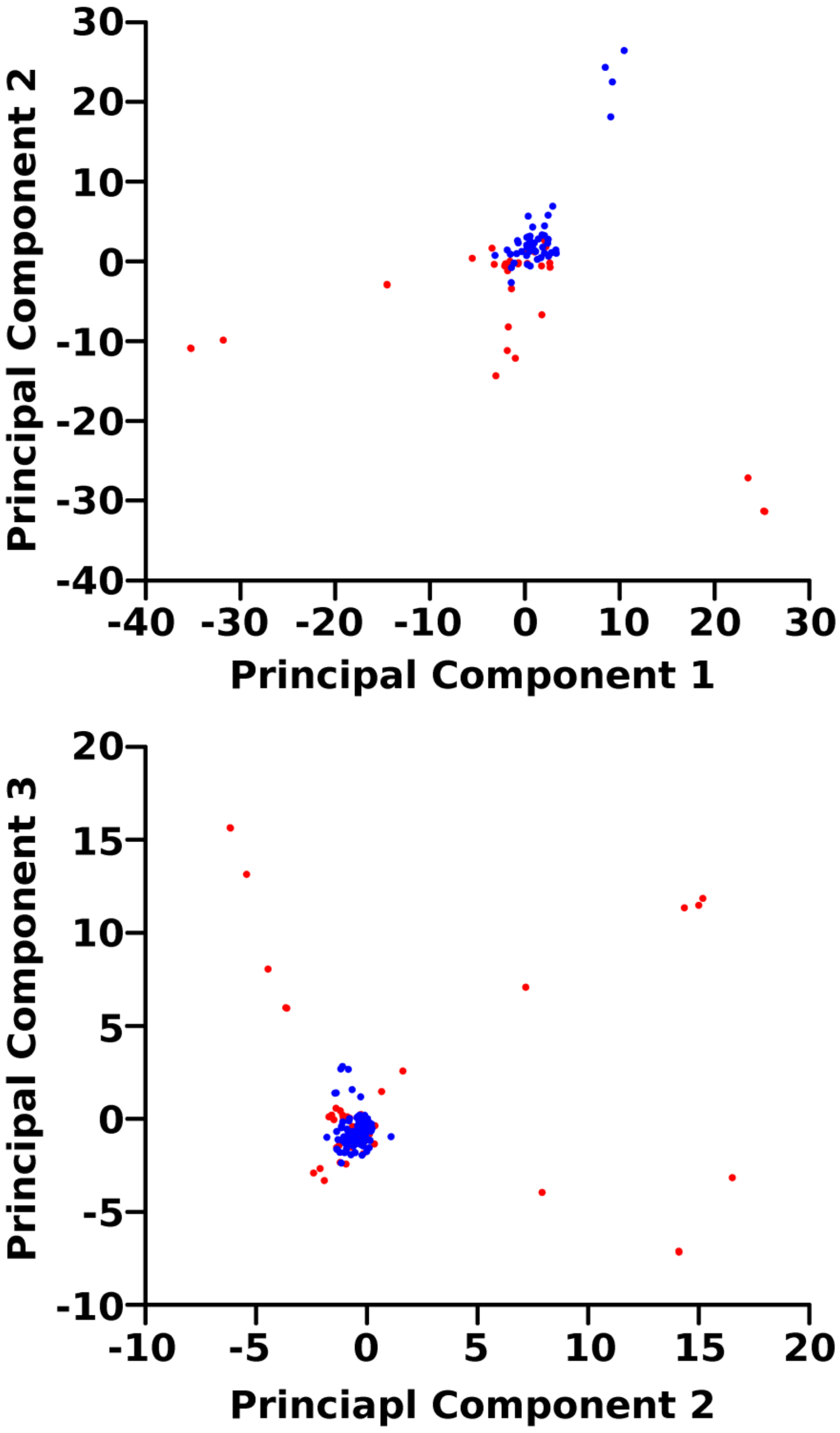

Supplementary Analysis Table I. 19 genes with Tajima's D scores of > 1 in both the Guinean and Gambian predominantly single genotype populations. Tajima's D scores were calculated for all genes with 3 or more SNPs following masking or repeat regions and exclusion of SNPs within introns.

Gene ID	Old Gene ID	Number of SNPs (Guinea)	Tajima's D (Guinea)	Number of SNPs (Gambia)	Tajima's D (Gambia)	Product Description
PF3D7_0113800	PFA0665w	223	1.28	207	1.00	DBL containing protein
PF3D7_0114500	PFA0700c	13	1.74	14	1.36	Plasmodium exported protein (hyp10)
PF3D7_0201600	PFB0080c	23	1.95	21	1.42	Plasmodium exported protein (PHISTb)
PF3D7_0221000	PFB0950w	21	1.42	17	2.29	Plasmodium exported protein
PF3D7_0321200	PFC0935c	17	1.60	14	1.92	N-acetylglucosamine-1-phosphate transferase, putative
PF3D7_0417800	PFD0865c	9	1.13	8	1.12	Cdc2-related protein kinase 1 (CRK1)
PF3D7_0420200	PFD0980w	15	1.56	13	1.76	Holo-(acyl-carrier protein) synthase
PF3D7_0422800	PFD1045w	13	1.29	13	1.03	Serpentine receptor, putative (SR12)
PF3D7_0508800	PFE0435c	4	1.53	3	1.01	Single-stranded DNA-binding protein (SSB)
PF3D7_0516300	PFE0815w	14	1.20	13	1.50	tRNA pseudouridine synthase
PF3D7_0710200	PF07_0042	119	1.44	108	1.69	Conserved Plasmodium protein
PF3D7_0710400	MAL7P1.32	8	1.39	8	1.01	Nucleotide excision repair protein
PF3D7_0720400	PF07_0085	11	1.01	11	1.32	Ferrodoxin reductase-like protein
PF3D7_1004800	PF10_0051	16	2.69	17	2.16	ADP/ATP carrier protein
PF3D7_1035700	PF10_0348	25	1.05	21	1.88	Duffy binding-like merozoite surface protein (DBLMSP)
PF3D7_1036300	PF10_0355	83	1.82	85	1.87	Merozoite surface protein (DBLMSP2)
PF3D7_1133400	PF11_0344	68	1.28	62	1.49	Apical membrane antigen 1 (AMA1)
PF3D7_1253100	PFL2555w	11	1.80	9	2.59	Plasmodium exported protein (PHISTa)
PF3D7_1301800	PF13_0075	138	1.15	119	1.43	Surface-associated interspersed protein 13.1 (SURFIN 13.1)

Supplementary Analysis Table II. Windows of directional selection for all *P. falciparum* genomic regions with at least 2 SNPs with |iHS| scores of > 3.29 (top 1% of expected distribution) for the Guinea predominantly single genotype population sample.

Chromosome	Window start (kb along chromosome)	Window end (kb along chromosome)	Region size (kb)	Number of SNPs	Genes within region
1	163	188	25	2	PF3D7_0103600 - PF3D7_0104200
2	367	552	184	2	PF3D7_0208900 - PF3D7_0213600
4	454	611	158	2	PF3D7_0409600 - PF3D7_0413300
**5**	**811**	**1034**	**222**	**2**	**PF3D7_0519600 - PF3D7_0524900**
6	1037	1285	249	22	PF3D7_0625600 - PF3D7_0630600
7	224	253	29	3	PF3D7_0704600 - PF3D7_0705100
7	254	317	63	4	PF3D7_0705100 - PF3D7_0706500
**7**	**338**	**518**	**180**	**9**	**PF3D7_0707300 - PF3D7_0711700**
7	611	629	9	4	PF3D7_0713400 - PF3D7_0713900
9	1180	1336	156	27	PF3D7_0929400 - PF3D7_0933800
9	1381	1437	56	2	PF3D7_0935500 - PF3D7_0936300
10	594	661	67	2	PF3D7_1014700 - PF3D7_1016400
10	1216	1552	336	12	PF3D7_1029900 - PF3D7_1038600
11	1270	1312	41	2	PF3D7_1132800 - PF3D7_1133800
12	693	1078	385	13	PF3D7_1217500 - PF3D7_1226600
12	1454	1996	542	7	PF3D7_1234800 - PF3D7_1248700
13	87	146	59	4	PF3D7_1301600 - PF3D7_1302700
13	304	367	63	2	PF3D7_1306500 - PF3D7_1308200
13	2420	2526	106	3	PF3D7_1360500 - PF3D7_1362800
14	1941	1992	51	2	PF3D7_1447500 - PF3D7_1448500

Bold, windows which overlap *mdr1* and *crt* on chromosomes 5 and 7 respectively.

Supplementary Analysis Table III. List of the most highly differentiated SNP allele frequencies between the Guinean and Gambian predominantly single genotype populations. F_{ST} scores were calculated for 106009 biallelic SNPs genome-wide (with a mean F_{ST} = 0.015).

Chromosome	SNP Position	Gene	Reference allele frequency (Guinea)	Reference allele frequency (Gambia)	F_{ST}	Coding effect	Amino Acid Change
7	375792	PF3D7_0708200	0.88	0.36	0.29	Synonymous	-
7	405600	PF3D7_0709000	0.78	0.30	0.23	Non-synonymous	I -> T
7	466461	PF3D7_0710200	0.30	0.76	0.20	Non-synonymous	N -> Y
7	466458	PF3D7_0710200	0.38	0.89	0.21	Non-synonymous	D -> E
7	466465	PF3D7_0710200	0.38	0.89	0.21	Non-synonymous	N -> Y
7	466466	PF3D7_0710200	0.38	0.89	0.21	Non-synonymous	N -> I
7	466482	PF3D7_0710200	0.38	0.89	0.21	Synonymous	-
9	413988	intergenic	0.88	0.44	0.23	-	-
9	775403	intergenic	1.00	0.68	0.23	-	-
9	1378602	PF3D7_0935400	0.60	0.09	0.26	Non-synonymous	P -> H
9	1382170	intergenic	0.76	0.15	0.36	-	-
9	1383344	intergenic	0.72	0.03	0.46	-	-
9	1384752	intergenic	0.74	0.18	0.30	-	-
9	1393934	intergenic	0.88	0.18	0.49	-	-
11	119497	PF3D7_1102500	0.96	0.61	0.20	Non-synonymous	S -> F

Supplementary Table IV. Tajima's D values genome-wide for all genes with 3 or more SNPs in the predominantly single genotype Guinean or Gambian populations. (Large EXCEL file given separately as part of Supplementary Table S3)

Supplementary Analysis Table V-A. Gene ontology terms significantly enriched amongst genes with a Tajima's D value of > 1.

GO ID	Term	Annotated	Significant	Expected	p-value
**Molecular function**					
**Guinea**					
GO:0004872	receptor activity	24	4	0.21	1.0×10^{-6}
GO:0016780	phosphotransferase activity for other substituted phosphate groups	5	2	0.05	8.9×10^{-4}
**Gambia**					
GO:0004872	receptor activity	23	4	0.30	6.3×10^{-5}
GO:0016780	phosphotransferase activity for other substituted phosphate groups	5	2	0.06	1.6×10^{-3}
**Biological process**					
**Guinea**					
GO:0009405	pathogenesis	17	5	0.21	1.0×10^{-6}
GO:0030260	entry into host cell	12	3	0.15	3.4×10^{-4}
**Gambia**					
GO:0009405	pathogenesis	17	5	0.25	2.2×10^{-6}
**Cellular compartment**					
**Guinea**					
GO:0016021	integral to membrane	306	9	3.35	3.6×10^{-3}

All terms that were highly significantly enriched ($p<0.01$) are shown for both predominantly single genotype populations. The total number of genes used in the analysis annotated with each term is shown along with the number of these genes that were significant (Tajima's D scores > 1). The number of genes expected to be significant given a random distribution is also shown. P-values calculated by Fisher's exact test and adjusted to account for the GO graph structure (Alexa et al 2006).

Supplementary Table V-B. Genes contributing to significantly enriched GO terms.

GO ID	Term	Gene ID	Product	Tajima's D Guinea	Tajima's D Gambia
GO:0004872	receptor activity	PF3D7_0113800	DBL containing protein, unknown function	1.28	1.00
		PF3D7_0424300	erythrocyte binding antigen-165, pseudogene (EBA165)	-0.40	1.02
		PF3D7_0731500	erythrocyte binding antigen-175 (EBA175)	1.01	0.95
		PF3D7_1035700	duffy binding-like merozoite surface protein (DBLMSP)	1.05	1.88
		PF3D7_1036300	merozoite surface protein (DBLMSP2)	1.82	1.87
GO:0016780	phosphotransferase activity for other substituted phosphate groups	PF3D7_0321200	N-acetylglucosamine-1-phosphate transferase, putative	1.60	1.92
		PF3D7_0420200	holo-(acyl-carrier protein) synthase, putative	1.56	1.76
GO:0009405	pathogenesis	PF3D7_0113800	DBL containing protein, unknown function	1.28	1.00
		PF3D7_0424300	erythrocyte binding antigen-165, pseudogene (EBA165)	-0.40	1.02
		PF3D7_0731500	erythrocyte binding antigen-175 (EBA175)	1.01	0.95
		PF3D7_1035700	duffy binding-like merozoite surface protein (DBLMSP)	1.05	1.88
		PF3D7_1036300	merozoite surface protein (DBLMSP2)	1.82	1.87
		PF3D7_1133400	apical membrane antigen 1 (AMA1)	1.28	1.49
GO:0030260	entry into host cell	PF3D7_0731500	erythrocyte binding antigen-175 (EBA175)	1.01	0.95
		PF3D7_1035400	merozoite surface protein 3 (MSP3)	1.11	0.72
		PF3D7_1133400	apical membrane antigen 1 (AMA1)	1.28	1.49
GO:0046812	integral to membrane	PF3D7_0104100	conserved Plasmodium membrane protein, unknown function	1.01	0.76
		PF3D7_0113800	DBL containing protein, unknown function	1.28	1.00

PF3D7_0114500	Plasmodium exported protein (hyp10), unknown function	1.74	1.36
PF3D7_0321200	N-acetylglucosamine-1-phosphate transferase, putative	1.60	1.92
PF3D7_0501300	skeleton-binding protein 1 (SBP1)	1.05	1.88
PF3D7_0731500	erythrocyte binding antigen-175 (EBA175)	1.01	0.95
PF3D7_1035700	duffy binding-like merozoite surface protein (DBLMSP)	1.05	1.88
PF3D7_1036300	merozoite surface protein (DBLMSP2)	1.82	1.87
PF3D7_1133400	apical membrane antigen 1 (AMA1)	1.28	1.49

For each GO term that is significantly enriched the associated genes that have Tajima's D scores above one in either population are shown along with their products and Tajima's D score in both the Guinean and Gambian population.

Supplementary Table VI. Linkage disequilibrium between the five high F_{ST} SNPs surrounding *gdv1* on *P. falciparum* chromosome 9. SNPs with significant linkage disequilibrium are highlighted in bold for each population sample.

	**Guinea**			**Gambia**		
**SNPs being compared**	D'	R^2	*p* value	D'	R^2	*p* value
1378602 / 1382170	0.306	0.044	0.192	0.214	0.026	0.401
1378602 / 1383344	**0.524**	**0.160**	**0.006**	0.468	0.219	0.059
1378602 / 1384752	**0.615**	**0.200**	**0.002**	0.185	0.015	0.475
1378602 / 1393934	**1.000**	**0.205**	**0.002**	0.185	0.015	0.466
1382170 / 1383344	**0.884**	**0.635**	**< 0.001**	1.000	0.174	0.159
1382170 / 1384752	**0.662**	**0.394**	**< 0.001**	0.022	0.000	0.546
1382170 / 1393934	**1.000**	**0.432**	**< 0.001**	**0.756**	**0.459**	**0.002**
1383344 / 1384752	**0.679**	**0.417**	**< 0.001**	0.389	0.044	0.175
1383344 / 1393934	**1.000**	**0.351**	**< 0.001**	1.000	0.140	0.197
1384752 / 1393934	**1.000**	**0.388**	**< 0.001**	-0.019	0.000	1.000
