# Supplementary material for: Genome-Wide Analysis of Selection on the Malaria Parasite Plasmodium falciparum in West African Populations of Differing Infection Endemicity
Source: Mol Biol Evol. 2014 Mar 18;31(6):1490–9. doi: 10.1093/molbev/msu106 (PMC4032133; doi:10.1093/molbev/msu106)

Supplementary Figure S1. Tajima's D value distribution is highest for genes predicted to have peak expression at the merozoite stage. Expression data for 3807 genes from microarray studies (Le Roch et al. 2003) retrieved from PlasmoDB (Aurrecoechea et al. 2009) were used to group genes by stage of peak expression. Points show the Tajima's D score for each gene in the Guinean population (grey bars show the medians for all genes with the same stage of peak expression) with predicted peak expression at each stage in the erythrocytic life cycle (ER, early ring; LR, late ring; ET, early trophozoite; LT, late trophozoite; ES, early schizont; LS, late schizont; M, merozoite). Asterisks indicate p values given by Mann-Whitney tests for the merozoite stage versus each other stage individually (* p<0.05, *** p<0.0001).

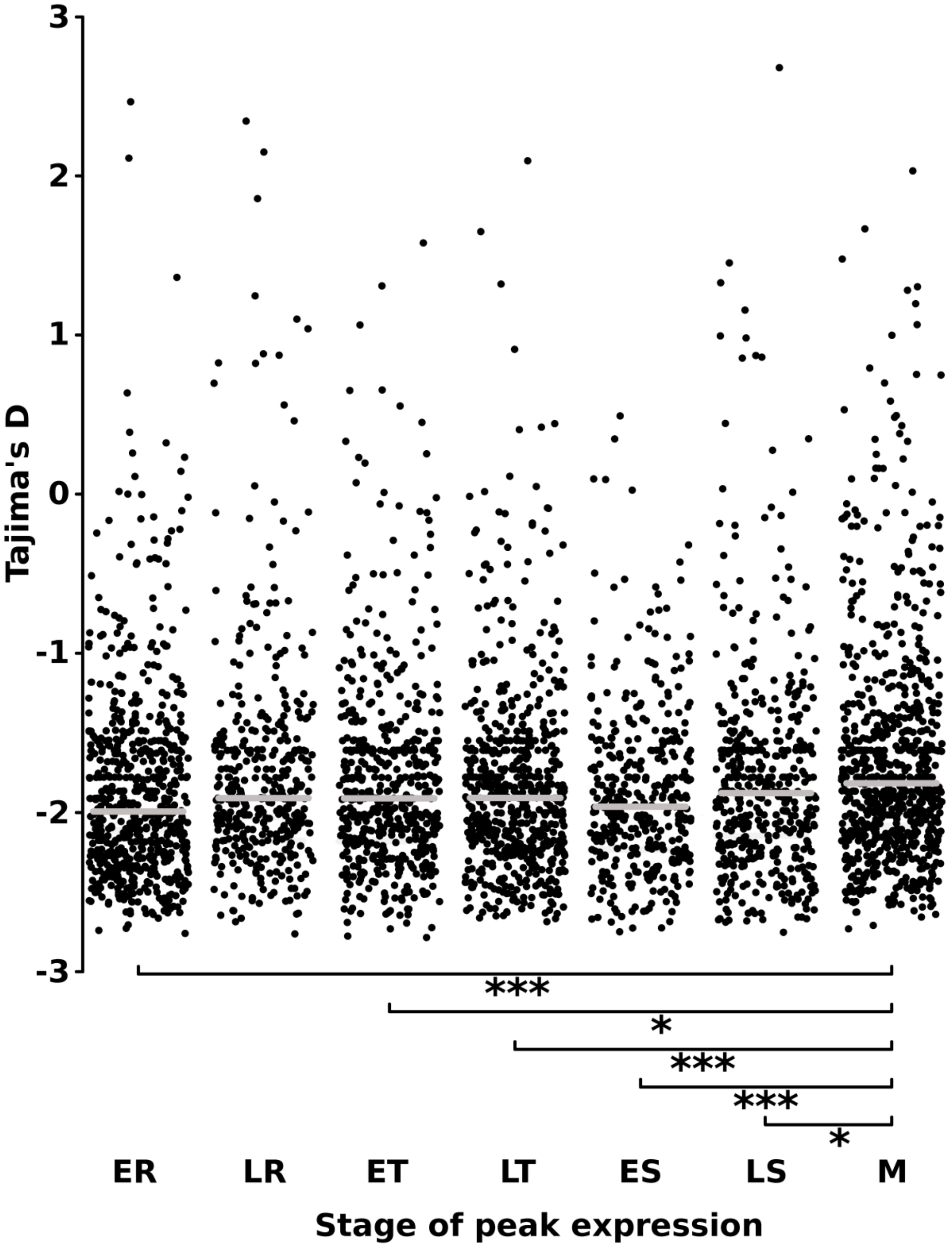

Supplementary Figure S2. Principal component analysis of 152 isolates from Guinea and Gambia utilising 112089 SNPs. A. First component and second component (3.2% and 2.8% of total variation respectively). B. Second and third component (2.6% of variation).

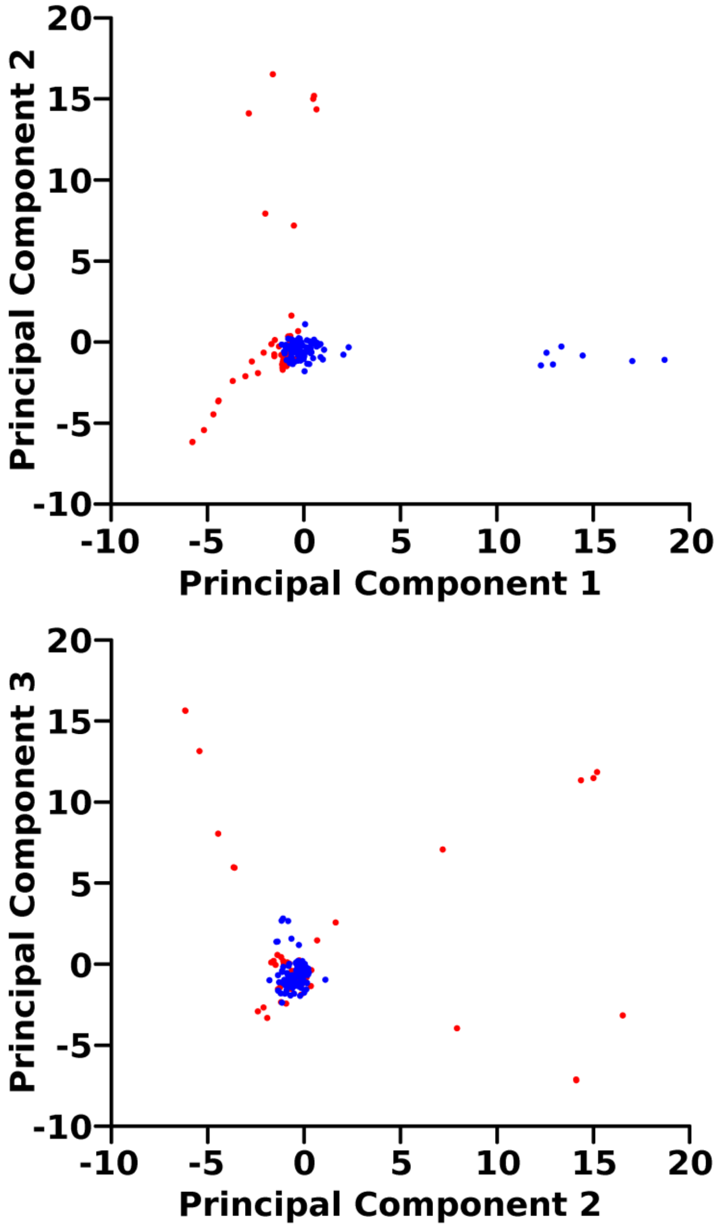

Supplement: Supplementary Data [file supp_msu106_Supplementary_Figures.pdf]
