# Supplementary material for: Genome-Wide Analysis of Selection on the Malaria Parasite Plasmodium falciparum in West African Populations of Differing Infection Endemicity
Source: Mol Biol Evol. 2014 Mar 18;31(6):1490–9. doi: 10.1093/molbev/msu106 (PMC4032133; doi:10.1093/molbev/msu106)
Supplement: Supplementary Data [file supp_msu106_Supplementary_Tables_S1-S2_S4-S7.pdf]

Supplementary Table S1. Isolate sequence read coverage and ENA reference numbers. [a] European Nucleotide Archive ID

Study ID	Sample Code	ENA ID[a]	Mean coverage (genome wide)
ZUC007	PA0245-C	ERS041967	99.1
ZUG116	PA0237-C	ERS042100	164.7
ZUG118	PA0152-C	ERS042063	27.9
ZRB072	PA0155-C	ERS042000	44.7
ZRB073	PA0231-C	ERS041991	45.8
ZRB078	PA0137-C	ERS042009	39.5
ZRB079	PA0148-C	ERS042054	128.6
ZRB242	PA0193-C	ERS042055	89.7
ZRB245	PA0190-C	ERS041981	24.5
ZRB613	PA0185-C	ERS042046	45.9
ZRB 615	PA0146-C	ERS041979	29.2
ZRB618	PA0215-C	ERS042069	34.8
ZRB619	PA0200-C	ERS042097	100.5
ZRB622	PA0216-C	ERS042102	159.0
ZRG313	PA0251-C	ERS042101	26.9
ZRG316	PA0138-C	ERS041976	83.9
ZRG323	PA0160-C	ERS042087	51.7
ZRG330	PA0253-C	ERS041978	24.4
ZRG349	PA0180-C	ERS041983	44.7
ZRG364	PA0179-C	ERS042089	75.4
ZRG365	PA0256-C	ERS042082	119.8
ZRG377	PA0163-C	ERS042065	33.6
ZRK276	PA0188-C	ERS042029	28.8
ZRK277	PA0182-C	ERS042073	20.3
ZRK282	PA0176-C	ERS042031	40.7
ZRL191	PA0227-C	ERS042075	105.8
ZRL205	PA0270-C	ERS042044	39.1
ZRL210	PA0263-C	ERS042084	45.2
ZRL211	PA0269-C	ERS042052	33.4
ZRL213	PA0267-C	ERS042013	16.0
ZRNs122	PA0232-C	ERS042028	35.4
ZRNs124	PA0243-C	ERS042012	27.0
ZRNs129	PA0249-C	ERS042036	36.7
ZRNs132	PA0239-C	ERS041993	40.9
ZRNs141	PA0134-C	ERS041972	112.4
ZRNs144	PA0233-C	ERS041994	41.5
ZRSa634	PA0261-C	ERS042066	18.3
ZRS042	PA0169-C	ERS042040	58.5
ZRS050	PA0153-C	ERS042074	141.8
ZRS066	PA0246-C	ERS041998	29.0
ZRS068	PA0222-C	ERS041992	39.8
ZRS715	PA0151-C	ERS042004	38.4

ZRS719	PA0225-C	ERS042045	44.4
ZRS736	PA0262-C	ERS042026	64.4
ZRSk161	PA0147-C	ERS042039	37.9
ZRSk162	PA0228-C	ERS041997	28.4
ZRSk184	PA0230-C	ERS042107	137.5
ZRSk185	PA0229-C	ERS042093	66.8
ZRSk744	PA0224-C	ERS042049	81.6
ZRY020	PA0184-C	ERS042010	38.6
ZRY027	PA0198-C	ERS041987	38.7
ZRY031	PA0255-C	ERS042003	14.6
ZRY033	PA0223-C	ERS042006	43.2
ZRY042	PA0234-C	ERS042106	60.4
ZRY298	PA0257-C	ERS042078	34.0
ZRY302	PA0178-C	ERS042090	117.7
ZRY304	PA0254-C	ERS041989	38.7
ZRY649	PA0210-C	ERS042111	113.8
ZRY657	PA0260-C	ERS042043	42.9
ZRY669	PA0214-C	ERS042002	29.2
ZUM378	PA0172-C	ERS041968	124.3
ZUM382	PA0165-C	ERS042079	147.6
ZUM390	PA0140-C	ERS041990	15.4
ZUM445	PA0149-C	ERS042067	19.8
ZUM448	PA0195-C	ERS042034	62.0
ZUM695	PA0221-C	ERS042071	76.5
ZUD403	PA0219-C	ERS042048	70.1
ZUD404	PA0197-C	ERS042032	44.7
ZUD414	PA0191-C	ERS042109	75.6
ZUD427	PA0189-C	ERS042112	128.0
ZUD428	PA0220-C	ERS041999	29.6
ZUD451	PA0183-C	ERS042062	35.3
ZUD458	PA0159-C	ERS042083	138.8
ZUD459	PA0187-C	ERS042104	151.3
ZUD464	PA0144-C	ERS042041	38.2
ZUD465	PA0168-C	ERS042038	79.8
ZUD468	PA0161-C	ERS041970	94.7
ZUD475	PA0157-C	ERS041975	41.4
ZUD478	PA0158-C	ERS042033	47.0
ZUD482	PA0171-C	ERS042001	52.6
ZUD488	PA0173-C	ERS042077	143.6
ZUD498	PA0167-C	ERS042108	128.5
ZUD499	PA0186-C	ERS042050	148.0
ZUD500	PA0145-C	ERS041974	132.0
ZUD501	PA0177-C	ERS042085	158.3
ZUD521	PA0208-C	ERS042088	140.2
ZUD534	PA0203-C	ERS041988	56.1
ZUD546	PA0209-C	ERS042099	160.8

ZUD548	PA0204-C	ERS042042	64.0
ZUD549	PA0201-C	ERS041986	42.6
ZUD566	PA0207-C	ERS042098	111.0
ZUD570	PA0217-C	ERS042070	72.4
ZUD573	PA0212-C	ERS042113	110.3
ZUD579	PA0211-C	ERS042110	149.6
ZUD581	PA0213-C	ERS042091	126.2
ZUD584	PA0206-C	ERS042056	133.0
ZUD585	PA0218-C	ERS042027	42.3
ZUH612	PA0205-C	ERS042105	76.8
ZUDk512	PA0199-C	ERS042086	128.1
ZUDk517	PA0202-C	ERS042057	124.7

Supplementary Table S2. F_{ws} scores for individual samples. Bold, samples where a single genotype dominated the infection (F_{ws} > 0.95. NA, this sample did not pass the quality controls for the F_{ws} analysis.

Population	Sample code	F_{ws}
Gambia	PA0008-C	0.38
	**PA0011-C**	**0.98**
	**PA0012-C**	**0.96**
	**PA0016-C**	**0.97**
	**PA0017-C**	**0.98**
	PA0018-C	NA
	**PA0020-C**	**0.97**
	PA0021-C	0.55
	**PA0022-C**	**0.97**
	PA0027-C	0.70
	**PA0029-C**	**0.96**
	PA0030-C	0.30
	**PA0032-C**	**0.96**
	**PA0034-C**	**0.96**
	**PA0035-C**	**0.96**
	**PA0036-C**	**0.96**
	**PA0037-C**	**0.96**
	**PA0038-C**	**0.97**
	PA0039-C	0.80
	PA0040-C	0.83
	PA0041-C	0.81
	**PA0042-C**	**0.97**
	PA0044-C	0.90
	**PA0045-C**	**0.97**
	**PA0049-C**	**0.96**
	**PA0050-C**	**0.95**
	PA0052-C	0.57
	PA0053-C	0.60
	**PA0057-C**	**0.95**
	**PA0060-C**	**0.98**
	PA0061-C	0.52
	**PA0065-C**	**0.97**
	**PA0066-C**	**0.97**
	**PA0067-C**	**0.98**
	PA0068-C	0.92
	PA0069-C	0.88
	**PA0071-C**	**0.98**
	**PA0074-C**	**0.97**
	**PA0075-C**	**0.97**

	**PA0078-C**	**0.96**
	**PA0081-C**	**0.97**
	PA0084-C	0.64
	PA0085-C	0.78
	**PA0091-C**	**0.95**
	PA0092-C	0.91
	**PA0093-C**	**0.97**
	**PA0097-C**	**0.95**
	PA0098-C	0.89
	**PA0099-C**	**0.98**
	**PA0100-C**	**0.96**
	**PA0101-C**	**0.97**
	**PA0102-C**	**0.96**
Guinea	**PA0134-C**	**0.95**
	**PA0137-C**	**0.99**
	PA0138-C	0.22
	PA0140-C	NA
	**PA0144-C**	**0.99**
	PA0145-C	0.52
	PA0146-C	0.41
	PA0147-C	0.37
	PA0148-C	0.86
	**PA0149-C**	**1.00**
	**PA0151-C**	**0.99**
	**PA0152-C**	**0.99**
	PA0153-C	0.72
	PA0155-C	0.41
	**PA0157-C**	**0.99**
	**PA0158-C**	**0.99**
	**PA0159-C**	**0.99**
	PA0160-C	0.41
	**PA0161-C**	**1.00**
	PA0163-C	0.63
	**PA0165-C**	**0.99**
	**PA0167-C**	**1.00**
	**PA0168-C**	**0.99**
	**PA0169-C**	**1.00**
	PA0171-C	0.73
	**PA0172-C**	**0.99**
	**PA0173-C**	**0.97**
	**PA0176-C**	**0.99**
	PA0177-C	0.85
	PA0178-C	0.80
	PA0179-C	0.61

	**PA0180-C**	**1.00**
	**PA0182-C**	**1.00**
	**PA0183-C**	**0.99**
	**PA0184-C**	**0.97**
	PA0185-C	0.85
	**PA0186-C**	**1.00**
	**PA0187-C**	**0.97**
	**PA0188-C**	**0.97**
	**PA0189-C**	**0.99**
	**PA0190-C**	**0.99**
	PA0191-C	0.48
	PA0193-C	0.80
	**PA0195-C**	**0.99**
	**PA0197-C**	**1.00**
	PA0198-C	0.86
	PA0199-C	0.86
	**PA0200-C**	**0.99**
	**PA0201-C**	**0.99**
	**PA0202-C**	**0.99**
	**PA0203-C**	**0.99**
	**PA0204-C**	**1.00**
	**PA0205-C**	**1.00**
	PA0206-C	0.80
	**PA0207-C**	**0.99**
	**PA0208-C**	**0.99**
	PA0209-C	0.84
	PA0210-C	0.43
	**PA0211-C**	**0.99**
	PA0212-C	0.71
	PA0213-C	0.89
	PA0214-C	0.89
	**PA0215-C**	**0.99**
	PA0216-C	0.82
	PA0217-C	0.66
	PA0218-C	0.63
	**PA0219-C**	**1.00**
	PA0220-C	0.52
	**PA0221-C**	**0.99**
	PA0222-C	0.25
	**PA0223-C**	**1.00**
	PA0224-C	0.39
	**PA0225-C**	**0.99**
	PA0227-C	0.52
	PA0228-C	0.48

	**PA0229-C**	**0.99**
	PA0230-C	0.36
	PA0231-C	0.25
	**PA0232-C**	**0.99**
	PA0233-C	0.18
	PA0234-C	0.36
	**PA0237-C**	**0.99**
	PA0239-C	0.26
	PA0243-C	0.34
	**PA0245-C**	**0.99**
	PA0246-C	0.84
	PA0249-C	0.33
	PA0251-C	0.82
	PA0253-C	0.39
	**PA0254-C**	**1.00**
	PA0255-C	NA
	**PA0256-C**	**0.99**
	PA0257-C	0.26
	**PA0260-C**	**0.97**
	PA0261-C	NA
	**PA0262-C**	**0.99**
	PA0263-C	0.53
	PA0267-C	NA
	PA0269-C	0.77
	**PA0270-C**	**0.99**

Supplementary Table S3. Tajima's D values genome-wide for all genes with 3 or more SNPs in Guinea or Gambia. (Large EXCEL file given separately)

Supplementary Table S4A. Gene ontology terms significantly enriched amongst genes with a Tajima's D value of > 1.

GO ID	Term	Annotated	Significant	Expected	p-value
**Molecular function**					
**Guinea**					
GO:0004872	receptor activity	24	3	0.15	3.5×10^{-4}
**Gambia**					
GO:0004872	receptor activity	24	4	0.25	7.8×10^{-5}
**Biological process**					
**Guinea**					
GO:0009405	pathogenesis	17	4	0.14	5.7×10^{-6}
**Gambia**					
GO:0009405	pathogenesis	17	5	0.25	2.2×10^{-6}
**Cellular compartment**					
**Gambia**					
GO:0016021	integral to membrane	288	10	4	3.9×10^{-3}

All terms that were highly significantly enriched (p<0.001) are shown for both populations. The total number of genes used in the analysis annotated with each term is shown along with the number of these genes that were significant (Tajima's D scores > 1). The number of genes expected to be significant given a random distribution is also shown. P-values calculated by Fisher's exact test and adjusted to account for the GO graph structure (Alexa et al 2006).

Supplementary Table S4B. Genes contributing to significantly enriched GO terms.

GO ID	Term	Gene ID	Product	Tajima's D Guinea	Tajima's D Gambia
GO:0004872	receptor activity	PF3D7_0113800	DBL containing protein, unknown function	1.36	1.15
		PF3D7_0731500	erythrocyte binding antigen-175 (EBA175)	0.58	1.22
		PF3D7_1035700	duffy binding-like merozoite surface protein (DBLMSP)	1.33	2.48
		PF3D7_1036300	merozoite surface protein (DBLMSP2)	2.68	2.50
GO:0009405	pathogenesis	PF3D7_0113800	DBL containing protein, unknown function	1.36	1.15
		PF3D7_0731500	erythrocyte binding antigen-175 (EBA175)	0.58	1.22
		PF3D7_1035700	duffy binding-like merozoite surface protein (DBLMSP)	1.33	2.48
		PF3D7_1036300	merozoite surface protein (DBLMSP2)	2.68	2.50
		PF3D7_1133400	apical membrane antigen 1 (AMA1)	1.45	1.70
GO:0046812	integral to membrane	PF3D7_0104100	conserved Plasmodium membrane protein, unknown function	1.16	1.05
		PF3D7_0113800	DBL containing protein, unknown function	1.36	1.15
		PF3D7_0114500	Plasmodium exported protein (hyp10), unknown function	1.04	1.27
		PF3D7_0321200	N-acetylglucosamine-1-phosphate transferase, putative	1.65	1.68
		PF3D7_0501300	skeleton-binding protein 1 (SBP1)	-	1.35
		PF3D7_0731500	erythrocyte binding antigen-175 (EBA175)	0.58	1.22
		PF3D7_1035700	duffy binding-like merozoite surface protein (DBLMSP)	1.33	2.48
		PF3D7_1036300	merozoite surface protein (DBLMSP2)	2.68	2.50
		PF3D7_1133400	apical membrane antigen 1 (AMA1)	1.45	1.70
		PF3D7_1204900	probable protein, unknown function	-1.57	1.13

Supplementary Table S5. All *P. falciparum* genomic regions with at least 2 SNPs with |iHS| scores of > 3.29 (top 1% of expected distribution) for the Guinea population sample.

Chromosome	Window start (kb along chromosome)	Window end (kb along chromosome)	Region size (kb)	Number of SNPs	Genes within region
1	163	184	21	4	PF3D7_0103600 - PF3D7_0104200
1	529	534	5	6	PF3D7_0113800 - PF3D7_0113800
2	324	552	227	2	PF3D7_020800 - PF3D7_0213600
3	142	216	74	3	PF3D7_0302600 - PF3D7_0304300
4	881	901	19	4	PF3D7_0419900 - PF3D7_0420000
4	1113	1131	19	2	PF3D7_0424600 - PF3D7_0425000
**5**	**808**	**1035**	**227**	**3**	**PF3D7_0519500 - PF3D7_0524900**
6	548	1275	727	28	PF3D7_0613500 - PF3D7_0630400
7	198	338	140	5	PF3D7_0704300 - PF3D7_0707200
**7**	**339**	**522**	**183**	**15**	**PF3D7_0707300 - PF3D7_0711700**
7	524	666	142	5	PF3D7_0711900 - PF3D7_0714500
7	1346	1374	28	2	PF3D7_0731300 - PF3D7_0731700
8	491	597	106	2	PF3D7_0809600 - PF3D7_0811900
9	1126	1419	293	16	PF3D7_0927700 - PF3D7_0935800
10	1208	1552	344	18	PF3D7_1029700 - PF3D7_1038600
11	1270	1318	48	4	PF3D7_1132800 - PF3D7_1133900
12	694	1095	401	10	PF3D7_1217600 - PF3D7_1227100
12	1454	2050	596	7	PF3D7_1234800 - PF3D7_1250100
13	93	193	101	8	PF3D7_1301600 - PF3D7_1303800

Supplementary Table S6. Linkage disequilibrium between the five high F_{ST} SNPs on *P. falciparum* chromosome 9. SNPs with significant linkage disequilibrium are highlighted in bold for each population sample.

	Guinea			Gambia		
**SNPs being compared**	D'	R^2	*p* value	D'	R^2	*p* value
1378602 / 1382170	**0.371**	**0.074**	**0.010**	0.071	0.003	0.664
1378602 / 1383344	**0.375**	**0.115**	**0.002**	0.432	0.119	0.066
1378602 / 1384752	**0.479**	**0.132**	**< 0.001**	0.136	0.014	0.585
1378602 / 1393934	**0.859**	**0.200**	**< 0.001**	0.111	0.008	0.609
1382170 / 1383344	**0.926**	**0.564**	**< 0.001**	**1.000**	**0.308**	**0.002**
1382170 / 1384752	**0.657**	**0.404**	**< 0.001**	0.278	0.054	0.198
1382170 / 1393934	**0.878**	**0.390**	**< 0.001**	**0.745**	**0.492**	**< 0.001**
1383344 / 1384752	**0.777**	**0.397**	**< 0.001**	**0.695**	**0.191**	**0.015**
1383344 / 1393934	**1.000**	**0.333**	**< 0.001**	**0.688**	**0.164**	**0.024**
1384752 / 1393934	**0.877**	**0.364**	**< 0.001**	0.309	0.084	0.061

Supplementary Table S7

Chromosome	End of 3' subtelomere	Start of 5' subtelomere
1	91652	565426
2	67544	860465
3	65507	1015545
4	170187	1150295
5	47760	1326241
6	39780	1329499
7	132430	1441012
8	58826	1349129
9	78873	1474420
10	61365	1599900
11	77999	2006704
12	56804	2190468
13	66918	2826696
14	28428	3266072
